# Stearoyl-CoA desaturase 1 regulates malignant progression of cervical cancer cells

**DOI:** 10.1080/21655979.2022.2079253

**Published:** 2022-05-24

**Authors:** Lingling Wang, Guoliu Ye, Yan Wang, Caizhi Wang

**Affiliations:** Department of Gynecology, The First Affiliated Hospital of Bengbu Medical College, Bengbu, Anhui, China

**Keywords:** Stearoyl-CoA desaturase 1, Kruppel like factor 9, epithelial-mesenchymal transition, cervical cancer, Akt/GSK3β

## Abstract

The primary regulatory gene for fatty acid synthesis, stearoyl-CoA desaturase 1 (SCD1), has been linked to the progression of several malignancies. Its role in cervical cancer remains unclear till now. This paper aimed to explore the role and mechanism of SCD1 in cervical cancer. The GEPIA database was used to perform a bioinformatics analysis of the role of SCD1 in cervical cancer staging and prognosis. The influences of SCD1 knockdown on cell proliferation, migration, invasion, and epithelial-mesenchymal transition (EMT) progress were then investigated. Following transcription factor Kruppel like factor 9 (KLF9) was discovered to be negatively correlated with SCD1, the regulatory role of KLF9 in the effects of SCD1 on cervical cancer cells and the signaling pathway was evaluated. According to the GEPIA database, SCD1 level was associated with the cervical cancer stage, the overall survival level, and the disease-free survival level. Cell proliferation, migration, invasion, and EMT progress were all hindered when its expression was knocked down. Novelty, KLF9 reversed the effects of SCD1 on cells, as well as the Akt/glycogen synthase kinase 3β (GSK3β) signaling pathway. Together, SCD1 was negatively regulated by KLF9 and it activated the Akt/GSK3β signaling pathway to promote the malignant progression of cervical cancer cells. Developing SCD1 inhibitors offers novel ideas for the biological treatment of cervical cancer.

## Graphic abstract

**Figure:**
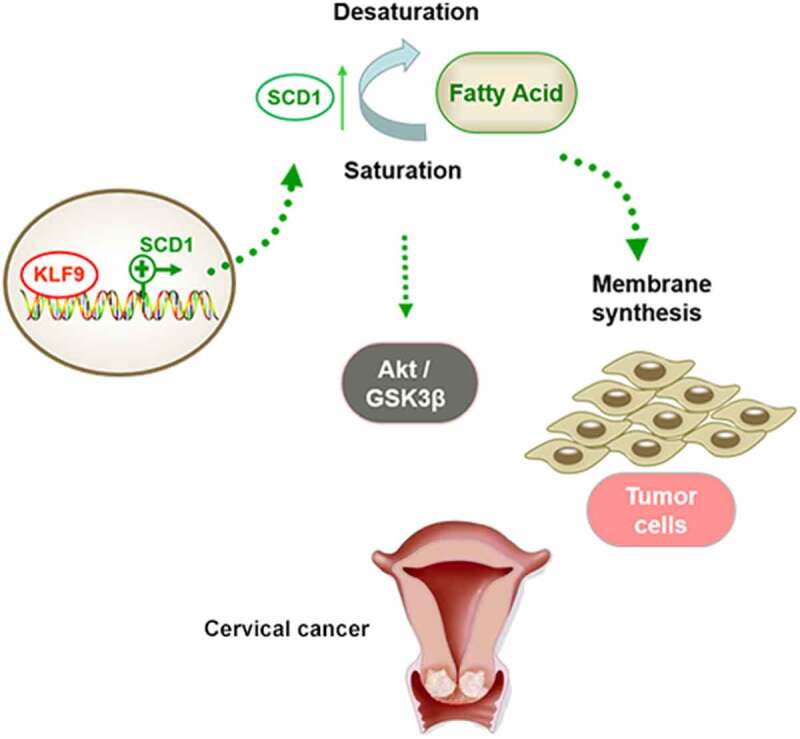

## Highlights

- SCD1 is up-regulated in cervical cancer tissue and cells
- KLF9 is negatively associated with SCD1
- SCD1 is regualted by KLF9 to affect cell malignant progression
- SCD1 activates the Akt/GSK3β signaling

## Introduction

Cervical cancer is one of the most familiar diagnosed gynecological malignancies [1]. According to statistics, generally, the morbidity and mortality rates vary greatly between nations, with China accounting for roughly one-sixth of the global cervical cancer burden [2]. Human papillomavirus (HPV) infection is the dominant cause of cervical carcinogenesis. Immune evasion of HPV can trigger uncontrolled proliferation of cervical epithelial cells, cervical tissue neoplasia, and ultimately invasive cancer [3]. It is delightful that cervical cancer vaccination can extremely prevent the occurrence of cervical cancer [4]. Nevertheless, due to the lack of adequate screening and HPV vaccination programs, the incidence in developing countries is six times higher than in developed countries [5]. Hence, these preventions are still required to be scaled up in developing countries. In addition to HPV, smoking, repeated pregnancies, and other risk factors can all contribute to the occurrence of cervical cancer [6]. Despite significant advances in diagnostic equipment and screening technologies, as well as significant progress in treatments and etiology research in recent years, cervical cancer remains an important threat to women’s lives and health [7]. Pathologically, tumor stromal infiltration and lymphatic metastasis are risk factors for the progress and prognosis of cervical cancer [8]. Cervical cancer is currently treated mostly with a combination of surgery and radiation therapy, with chemotherapy thrown in for good measure [9]. Notably, gene therapy and immunotherapy have obtained increasing attention as molecular biology has progressed [10,11].

Stearoyl-CoA desaturase 1 (SCD1) is a central regulator that controls cell metabolism and cell cycle progression. It is a crucial regulator of fatty acid synthesis and a catalyst for the conversion of saturated to monounsaturated fatty acids [12]. SCD1 is essential for catalyzing membrane biogenesis and is extensively involved in lipid metabolism, allowing cells to continue to proliferate [13]. Previous studies have indicated that the high expression of SCD1 is associated with the pathogenesis of various diseases. In addition to obesity, diabetes, hyperlipidemia, it has been confirmed that SCD1 also participates in the onset and advancement of several malignancies [14]. For instance, it has been reported that SCD1 has the capacity to enhance gastric tumor growth, migration, and anti-ferroptosis, and high expression of SCD1 may predict poor prognosis in patients with gastric cancer [15]. Upregulation of SCD1 expression was also observed in lung cancer cell lines, and SCD1 overexpression inhibited apoptosis in gefitinib-treated cells [16]. Moreover, SCD1 was found to be highly expressed in colon cancer tissues and negatively correlated with prognosis, and SCD1 increased the progression of colon cancer by promoting epithelial-mesenchymal transition (EMT) [17]. In addition, there was a significant correlation between SCD1 expression and poor prognosis in patients with bladder cancer, and SCD1 inhibitor markedly reduced cell proliferation and invasion [18]. However, no information on the mechanism of SCD1 in cervical cancer has been published. According to the Gene Expression Profiling Interactive Analysis (GEPIA) database, SCD1 is up-regulated in cervical cancer tissues and is associated with a poor prognosis.

As a result, early in the project, bioinformatics analysis of the role of SCD1 in cervical cancer was carried out, and afterward, cervical cancer cell lines were used for experimental investigation. This paper aimed to explore the involvement and mechanism of SCD1 in cervical cancer cell lines. The findings support the idea that SCD1 could be used as a therapeutic target for cervical cancer.

## Materials and methods

### Bioinformatics analysis

GEPIA database (gepia.cancer-pku.cn) contains the RNA sequencing expression data of tumors and normal samples from the TCGA and the GTEx projects [19]. The expression level of SCD1 in cervical cancer tissue, and the association between its level and stage, the overall survival level, and the disease-free survival level were analyzed through GEPIA. The expression level of Kruppel like factor 9 (KLF9) in cervical cancer tissue, and the association between its level and stage, SCD1 expression were analyzed. JASPAR database (jaspar.genereg.net) contains information about transcription factor binding motif, which can be used to predict the binding region of transcription factors and sequences [20]. The binding site between KLF9 and SCD1 promoter was predicted through the JASPAR database.

### Cell culture

Human cervical squamous cells Ect1/E6E7 and cervical cancer cell lines siHa, HeLa, C33A, CaSki, and HCC94 were obtained from Fenghui Biotechnology (Changsha, Hunan, China). They were cultured in Roswell Park Memorial Institute (RPMI)-1640 (Sartorius, Shanghai, China) containing 10% fetal bovine serum (FBS; Merck, USA) and 1% penicillin/streptomycin (Gibco) at 37°C and 5% CO_2_ [21].

### Cell transfection

Cells were transfected with FuGENE HD reagent (Roche, Shanghai) [22]. Short hairpin RNA (shRNA) targeting SCD1 for precise knockdown (shRNA-SCD1-1/2) and scrambled shRNA as the negative control (shRNA-NC) were used. Otherwise, shRNA targeting KLF9 (sh-KLF9-1/2) or pcDNA3.1 vector containing KLF9 (Oe-KLF9) for knockdown or overexpression were used along with NCs separately named sh-NC and Oe-NC. They were all obtained from GenePharma (Shanghai, China).

### Reverse transcription-quantitative PCR (RT-qPCR)

Total RNA was extracted and complementary DNA was acquired with TRIzol® reagent (Invitrogen) and PrimeScript RT reagent kit (Takara, Beijing, China). RT-qPCR was conducted with the QuantiTect SYBR Green PCR kit (Qiagen). The relative mRNA levels were quantified using the ∆∆Cq method [23] following normalization against GAPDH. Used primers are listed in Table 1.

**Table 1.** Primer sequences used for RT-qPCR analysis.

Gene	Sequence (5’→ 3’)
SCD1	Forward CTTGCGATATGCTGTGGTGC
	Reverse AGGGGAAGGAGTGAAAGGGA
KLF9	Forward AGAAGAGGCACACTTGACGG
	Reverse GGGACCGAGTGTTGTTGACT
GAPDH	Forward GACTCATGACCACAGTCCATGC
	Reverse AGAGGCAGGGATGATGTTCTG

### Western blotting

Protein were extracted from cells following radio-immunoprecipitation assay (RIPA) lysis buffer (Solarbio, Beijing, China) treatment and quantified using a bicinchoninic acid assay (BCA; Solarbio). The proteins were apart through SDS-PAGE on the polyacrylamide gel and shifted to polyvinylidene fluoride (PVDF) membranes (Roche). The membranes were incubated with skimmed milk, primary antibodies, and horse radish peroxidase (HRP)-conjugated antibody in sequence. After blots were visualized with an enhanced chemiluminescence (ECL) detection reagent (Millipore), data were analyzed with ImageJ 1.52 [24]. Used antibodies are presented in Table 2.

**Table 2.** Antibodies used in western blotting.

Antibody	Catalog number	Host	Dilution ratio	Company
SCD1	A305-259A	Rabbit	1:5,000	ThermoFisher
MMP2	10,373-2-AP	Rabbit	1:5,000	ThermoFisher
MMP9	ab283575	Rabbit	1:1,000	Abcam
Ki67	ab16667	Rabbit	1:1,000	Abcam
PCNA	ab29	Rabbit	1:1,000	Abcam
E-cadherin	GTX100443	Rabbit	1:5,000	GeneTex
N-cadherin	GTX127345	Rabbit	1:2,000	GeneTex
Vimentin	GTX100619	Rabbit	1:1,0000	GeneTex
Snail	GTX125918	Rabbit	1:2,000	GeneTex
KLF9	ab227920	Rabbit	1:1,000	Abcam
p-Akt	GTX128414	Rabbit	1:2,000	GeneTex
Akt	GTX121936	Rabbit	1:5,000	GeneTex
P-GSK3β	Ab68476	Rabbit	1:1,000	Abcam
GSK3β	AF5174	Rabbit	1:2,000	Beyotime
GAPDH	AF1186	Rabbit	1:2,000	Beyotime
HRP anti-rabbit lgG	A0208	goat	1:1,000	Beyotime

### Cell counting kit-8 (CCK-8) assay

Cells (5 × 10^3^ cells/well) were seeded in a 96-well plate and separately incubated for 24, 48, and 72 h [25]. For each time point, the wells were supplemented with 10 µl of CCK-8 solution (Dojindo). 3 h later, absorbance was acquired with a microplate reader (450 nm, Perlong, Beijing).

### Colony formation assay

Cells (500 cells/dish) were seeded into culture dishes and cultured for 2 weeks. At the last day, the cells were fixed with 4% paraformaldehyde for 15 min. The fixative solution was then altered with crystal violet (Yeasen, Shanghai, China) to staining for 30 min. A cluster > 50 cells was considered as a colony [26].

### Cell migration and invasion

The migration and invasion was separately assessed with wound healing and Transwell assays [27]. Cells were cultured until a confluent monolayer formed and sterile pipette tip was used to generate a wound in the middle. Images were captured at 0 and 24 h. For Transwell assay, cells were cultivated in the upper chamber which was pre-coated with Matrigel (Corning). Meanwhile, RPMI-1640 containing 20% FBS was loaded in the lower chamber. Following 24 h of incubation, the invasive cells were fixed and stained. Results were both observed under a microscope (x100; Olympus).

### Luciferase reporter assay

The SCD1 promoter region, spanning from −2000 to +100 of the transcription start site or mutant sequence, was cloned into the pGL3-Basic vector [28]. The mixture of the luciferase reporter plasmid and the Oe-KLF9 was co-transfected into cells using FuGENE HD. Cells were harvested following transfection for 24 h, and then the luciferase activity was measured by means of the luciferase reporter system (Promega, USA).

### Chromatin immunoprecipitation (ChIP)

The binding between KLF9 and SCD1 was determined using a ChIP assay (Beyotime. Nantong, China) [29]. The cells were cross-linked with methanol and terminated with glycine, followed by lysis and sonication. Partial supernatant was as Input, while others were incubated with an anti-KLF9 antibody overnight. Following the incubation, beads were added to harvest the protein-DNA complex and 5 M sodium chloride (NaCl) was added to retrieve the DNA. The enrichment was determined using RT-qPCR as described above.

### Statistical analysis

Experiments were independently conducted three times. The data are presented as the mean ± standard deviation (SD) and statistical analysis was performed with Prism 8.0 software. Student t test along with one-way analysis of variance (ANOVA) followed by Tukey’s post hoc test [30] was separately used to compare the differences between two and multiple groups. P < 0.05 indicates a significant difference.

## Results

Endogenous fatty acid synthesis has been found to play a tumor-promoting role in malignant tumors. Up to now, the mechanism of SCD1, an enzyme that mediates fatty acid synthesis, in cervical cancer remains undisclosed. The purpose of the present study was to explore the role and mechanism of SCD1 in cervical cancer. According to the GEPIA database, SCD1 level was found associated with the cervical cancer stage, the overall survival level, and the disease-free survival level. In addition, knocking down its expression inhibited cell proliferation, migration, invasion, and EMT progress. KLF9 knockdown reversed the effects of SCD1 knockdown on cells, as well as the Akt/glycogen synthase kinase 3β (GSK3β) signaling pathway, indicating SCD1 was negatively regulated by KLF9.

### SCD1 levels in cervical cancer tissue and cell lines

**Figure 1.**
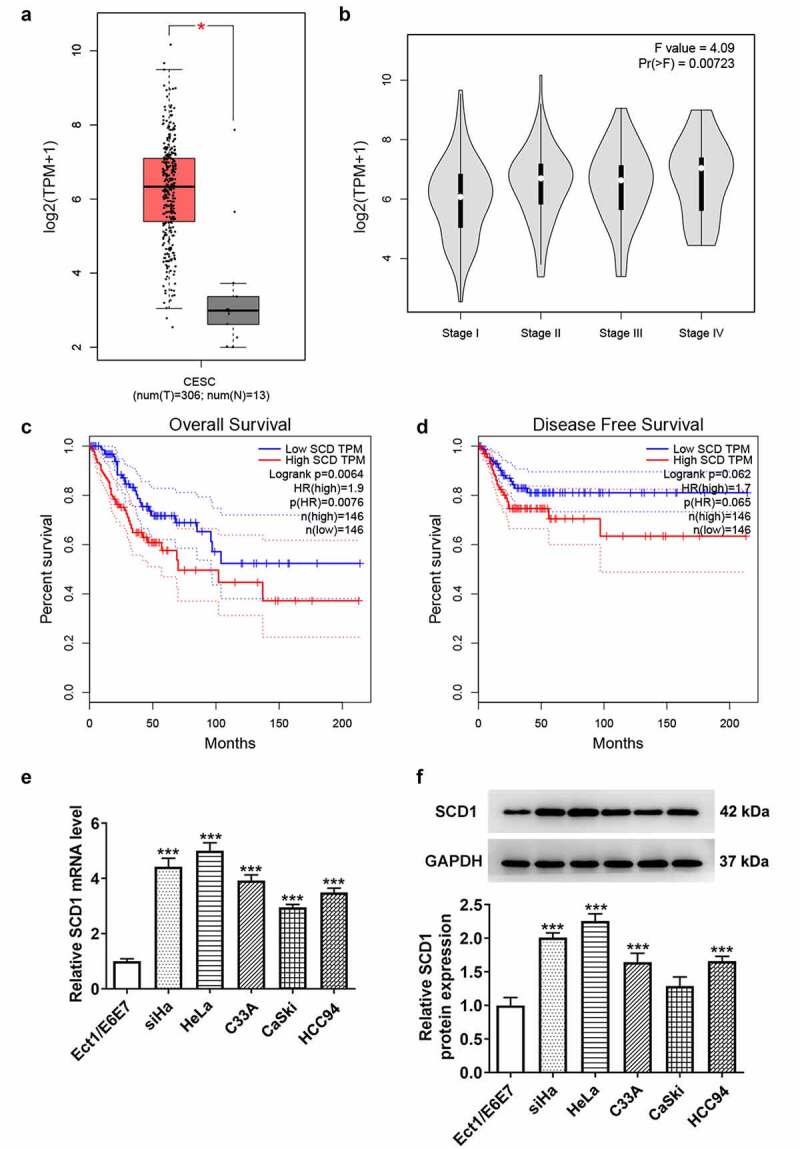
The expression levels of SCD1 in cervical cancer tissue and cell lines (a) The GEPIA database was used to analyze the expression level of SCD1 in cervical cancer tissue samples, (b) the association between SCD1 expression level and stage, (c) the overall survival level, (d) and the disease-free survival level. (e) The expression level of SCD1 in cervical cancer cells was determined using RT-qPCR and (f) western blotting. n = 3; ***P < 0.001 vs. Ect1/E6E7.

The roles of SCD1 in cervical cancer were analyzed through the GEPIA database. A total of 306 cervical cancer tissue samples and 13 normal samples were included. The expression level of SCD1 was significantly up-regulated in cervical cancer tissue samples (Figure 1(a)). The association between SCD1 expression level and stage (Figure 1(b)), the overall survival level (Figure 1(c)), and the disease-free survival level (Figure 1(d)) in cervical cancer was then analyzed. The expression of SCD1 was higher in advanced cervical cancer, and its high expression was positively correlated with poor prognosis. Subsequently, the expression level of SCD1 in cervical cancer cells was determined using RT-qPCR and western blotting (Figure 1(e-f)). Its expression was found to be up-regulated in siHa, HeLa, C33A, CaSki, and HCC94 cells compared with Ect1/E6E7 cells. To highlight the role of SCD1 expression in cells, HeLa cells expressing the highest SCD1 levels were selected.

### SCD1 knockdown inhibits the malignant progress

**Figure 2.**
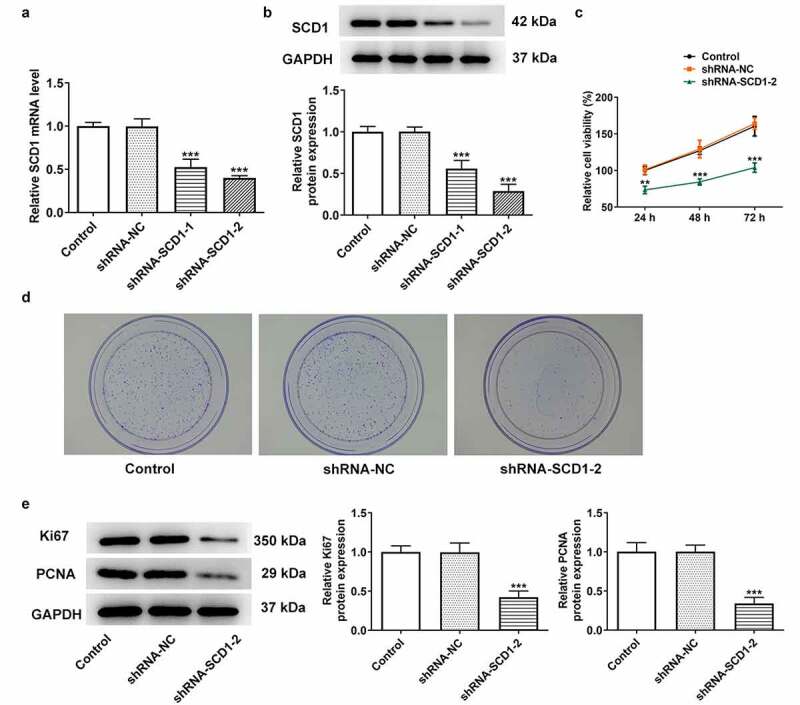
SCD1 knockdown inhibits cell proliferation (a) Cells were transfected with shRNA-SCD1, and the efficacy was assessed using RT-qPCR and (b) western blotting. (c) Cell proliferation was determined using a CCK8 assay. (d) The colony-forming ability of cells was assessed using colony formation assay. (e) The expression levels of proliferation-related proteins were determined using western blotting. n = 3; ***P < 0.001 vs. shRNA-NC.

HeLa cells were transfected with shRNA-SCD1, and the efficacy was assessed using RT-qPCR and western blotting (Figure 2(a-b)). The cells in the shRNA-SCD1-2 group were selected for the following assays due to the lower level of SCD1. Cell proliferation was determined using a CCK8 assay (Figure 2(c)). SCD1 knockdown was found to decline the proliferation of cells. In addition, the colony-forming ability of cells was assessed using colony formation assay (Figure 2(d)). The formative colony in the shRNA-SCD1-2 group was obviously less than that in the shRNA-NC group. The expression levels of proliferation-related proteins were determined using western blotting (Figure 2e). Ki67 and PCNA expression levels were both decreased in the shRNA-SCD1-2 group.

**Figure 3.**
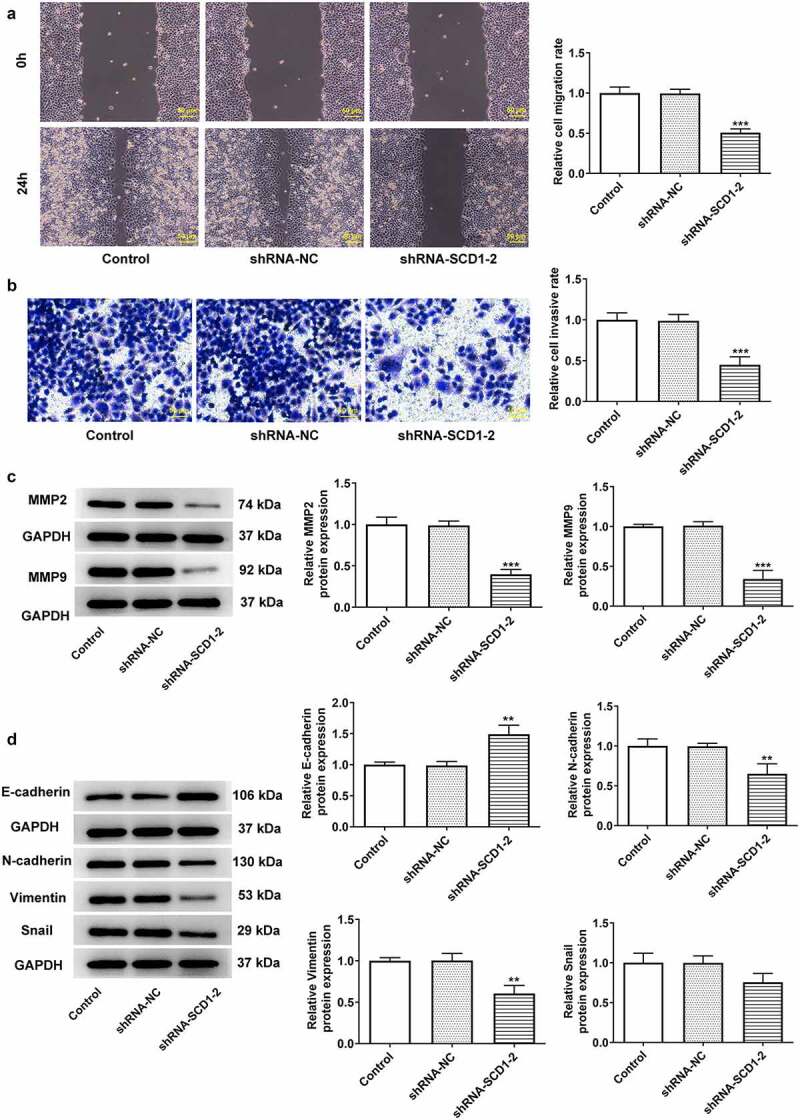
SCD1 knockdown inhibits cell migration, invasion, and EMT progress (a) Cell migration and (b) invasion was determined using wound healing and Transwell assay, respectively. (c) The expression level of MMP2 and MMP9 was determined using western blotting. (d) The expression level of EMT-related protein was determined using western blotting. n = 3; **P < 0.01, ***P < 0.001 vs. shRNA-NC.

Afterward, cell migration and invasion were determined using wound healing and Transwell assays, respectively (Figure 3(a-b)). The results indicated that SCD1 knockdown significantly inhibited cell migration and invasion abilities. The expression level of matrix metalloproteinase (MMP)2 and MMP9 was also declined according to the result of western blotting (Figure 3(c)). Subsequently, the expression level of EMT-related protein was determined using western blotting (Figure 3(d)). The expression level of E-cadherin was elevated due to the down-regulated SCD1 expression, whereas that of N-cadherin, Vimentin, and SNAIL was decreased.

### The association between KLF9 and SCD1

**Figure 4.**
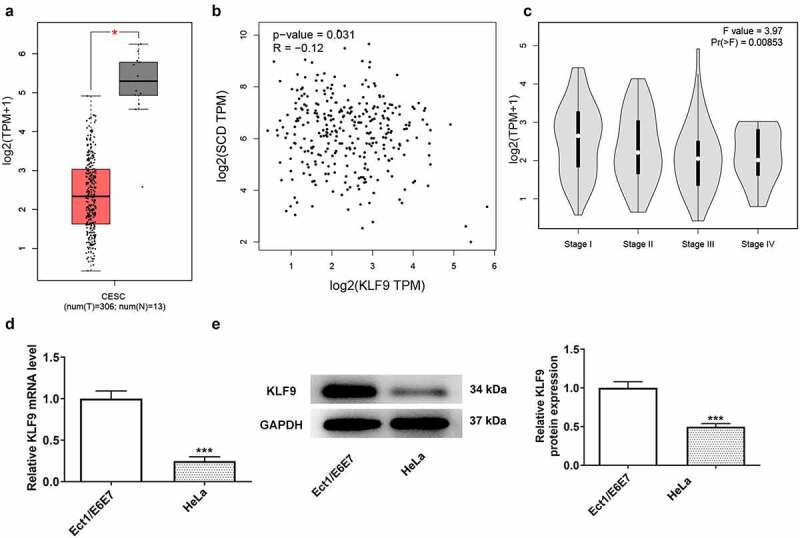
The role of KLF9 in cervical cancer (a) The GEPIA database was used to analyze the expression level of KLF9 in cervical cancer tissue samples, (b) the association between KLF9 expression level and stage, (c) and SCD1. (d) The expression level of KLF9 in HeLa cells was determined using RT-qPCR and (e) western blotting. n = 3; ***P < 0.001 vs. Ect1/E6E7.

The expression level of KLF9 was down-regulated in cervical cancer tissues in accordance with the GEPIA database (Figure 4(a)). The lower expression of KLF9 was found in advanced cervical cancer (Figure 4(b)) and negatively correlated with SCD1 (Figure 4(c)). Hence, the expression level of KLF9 in HeLa cells was determined using RT-qPCR and western blotting (Figure 4(d-e)). Its expression level was significantly descended in HeLa cells compared with Ect1/E6E7 cells.

**Figure 5.**
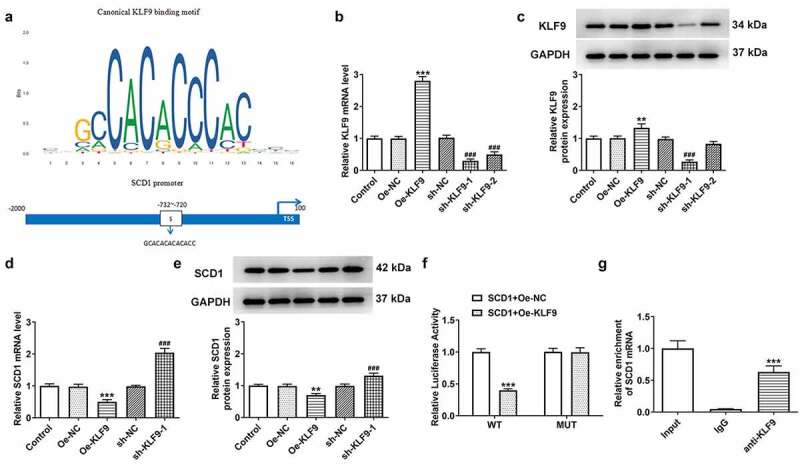
The association between KLF9 and SCD1 (a) The binding site between KLF9 and SCD1 promoter was predicted through the JASPAR database. (b) The efficacy of KLF9 overexpression and knockdown was evaluated using RT-qPCR and (c) western blotting. (d) The expression level of SCD1 in these cells was detected using RT-qPCR and (e) western blotting. n = 3; **P < 0.01, ***P < 0.001 vs. Oe-NC; ^###^P < 0.001 vs. sh-NC. (f) The activity of the SCD1 promoter was measured with a luciferase reporter assay. n = 3; ***P < 0.001 vs. SCD1-WT + Oe-KLF9. (g) The combination between KLF9 and SCD1 promoter was assessed using a ChIP assay. n = 3; ***P < 0.001 vs. lgG.

The binding site between KLF9 and SCD1 promoter predicted through the JASPAR database was displayed in Figure 5(a). The efficacy of KLF9 overexpression and knockdown was evaluated using RT-qPCR and western blotting, respectively (Figure 5(b-c)). The cells in the Oe-KLF9 group were effectively overexpressing KLF9. Moreover, the cells in the sh-KLF9-1 group were selected for the following assays resulting from the lower KLF9 expression level in comparison with the sh-KLF9-2 group. The expression level of SCD1 in these cells was detected with RT-qPCR and western blotting (Figure 5(d-e)). The SCD1 expression level was lower in KLF9-overexpressing cells, whereas SCD1 levels were elevated in KLF9-knockdown cells. The activity of SCD1 promotor was measured with a luciferase reporter assay, and the activity of luciferase was found to be down-regulated in the SCD1-WT + Oe-KLF9 group (figure 5(f)). Furthermore, the combination between KLF9 and SCD1 promoter was assessed using a ChIP assay (Figure 5(g)). The enrichment of SCD1 was detected in the anti-KLF9 group, whereas there was no enrichment in the lgG group.

### KLF9 influences the regulations of SCD1

**Figure 6.**
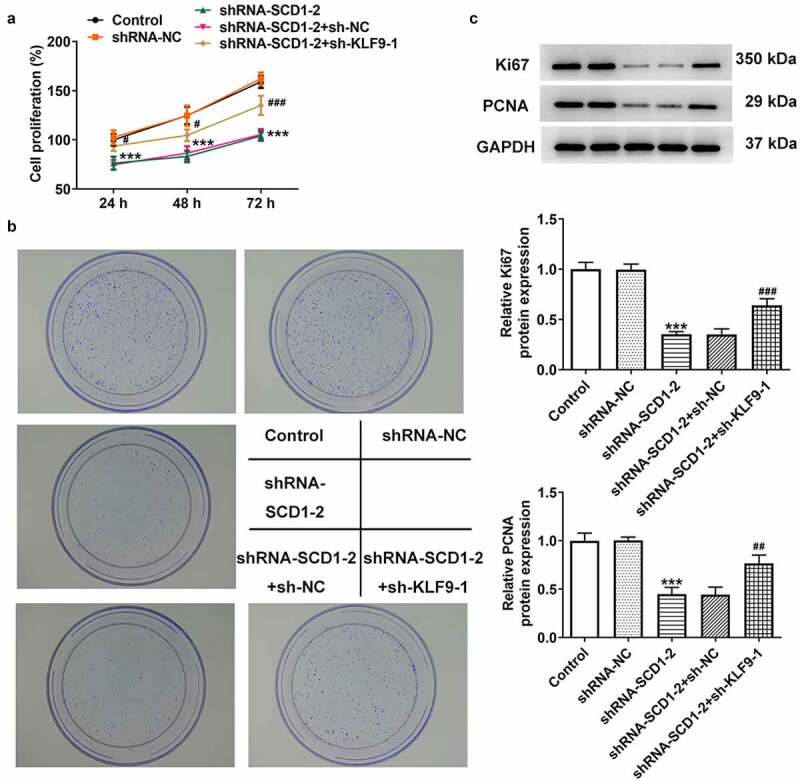
KLF9 knockdown reverses the impacts of SCD1 knockdown on cell proliferation (a) The proliferation level of cells co-transfected with shRNA-SCD1-2 and sh-KLF9-1 was assessed using a CCK8 assay. (b) The colony-forming ability was assessed using a colony formation assay. (c) The expression levels of proliferation-related proteins were determined using western blotting. n = 3; ***P < 0.001 vs. shRNA-NC; ^#^P < 0.05, ^##^P < 0.01, ^###^P < 0.001 vs. shRNA-SCD1-2 + sh-NC.

**Figure 7.**
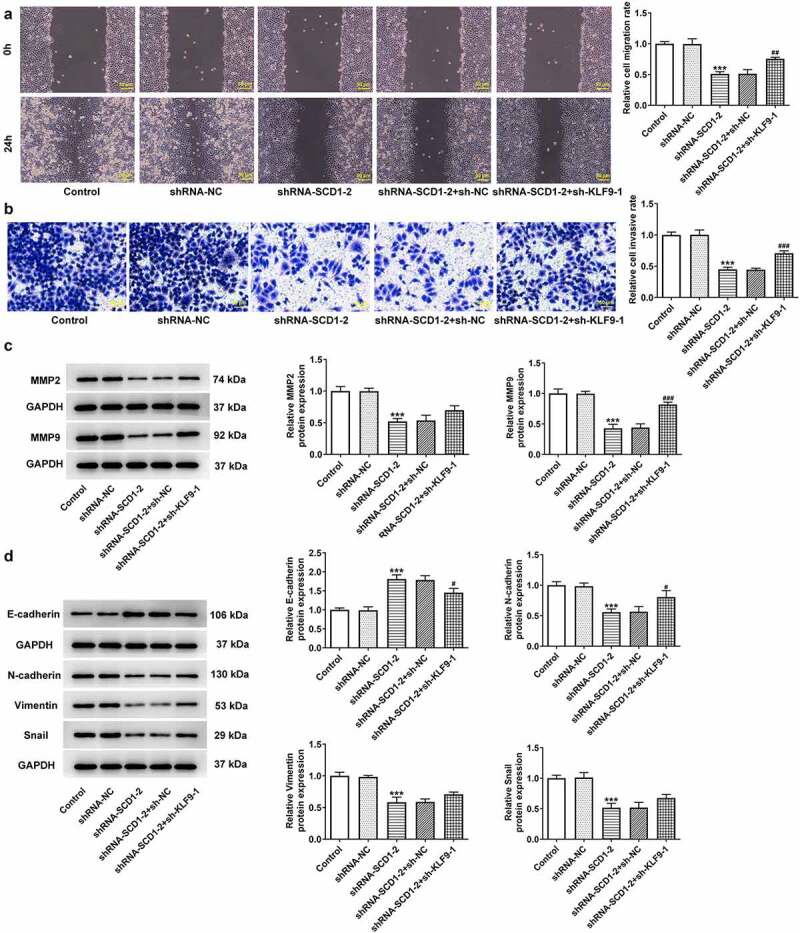
KLF9 knockdown reverses the impacts of SCD1 knockdown on cell migration, invasion, and EMT progress (a) Cell migration and (b) invasion was determined using wound healing and Transwell assay, respectively. (c) The expression level of MMP2 and MMP9 was determined using western blotting. (d) The expression level of EMT-related protein was determined using western blotting. n = 3; ***P < 0.001 vs. shRNA-NC; ^#^P < 0.05, ^##^P < 0.01, ^###^P < 0.001 vs. shRNA-SCD1-2 + sh-NC.

**Figure 8.**
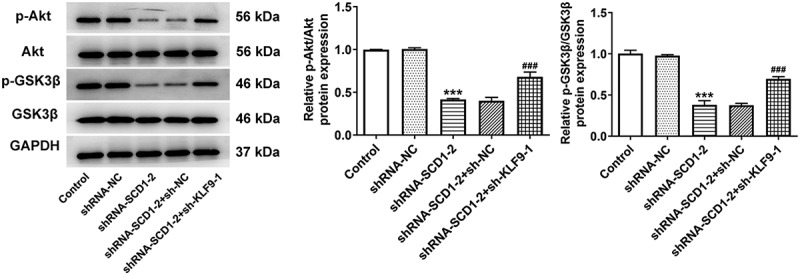
KLF9 knockdown reverses the impacts of SCD1 knockdown on the Akt/GSK3β signaling The expression levels of proteins related to the Akt/GSK3β were determined using western blotting. n = 3; ***P < 0.001 vs. shRNA-NC; ^###^P < 0.001 vs. shRNA-SCD1-2 + sh-NC.

Following cells were co-transfected with shRNA-SCD1-2 and sh-KLF9-1, the proliferation level (Figure 6(a)) and the colony-forming ability (Figure 6(b)) were assessed. The results of the CCK8 and colony formation assays indicated that KLF9 knockdown reversed the reduced cell proliferation and colony-forming ability caused by SCD1 knockdown. The results of western blotting revealed that KLF9 knockdown partly elevated the expression level of Ki67 and PCNA (Figure 6(c)). In addition, KLF9 knockdown likewise increased cell migration and invasion according to the results of wound healing and Transwell assays (Figure 7(a-b)). The results of western blotting revealed that the expression levels of MMP2, MMP9, N-cadherin, Vimentin, and SNAIL were up-regulated as a consequence of KLF9 knockdown (Figure 7(c-d)). Moreover, the expression levels of proteins related to Akt/GSK3β were determined using western blotting (Figure 8). The expression levels of p-Akt and p-GSK3β were declined in the shRNA-SCD1-2 group and partly ascended in the shRNA-SCD1-2 + sh-KLF9-1 group.

## Discussion

Despite advances in surgery plus radiotherapy and chemotherapy, partial patients with cervical cancer still experience early metastases from the primary tumor, especially lymph node metastases, which lead to poor prognosis and poor treatment outcomes [31]. EMT refers to the morphological transition of polarized epithelial cells to locomotor mesenchymal cells [32], which is manifested by the descending expression of E-cadherin and keratin, the elevated expression of vimentin, N-cadherin, and MMPs, and the migration of cells is enhanced [33]. EMT process is implicated in regulating cancer cell invasion in numerous studies [34]. The tumor microenvironment, hypoxia, and inflammation influence the expression of EMT-related transcription factors (such as SNAIL and TWIST) and cadherin to govern tumor cell metastasis [35–38]. Importantly, HPV can directly induce EMT, and EMT markers have been found to alter during cancer metastasis [39]. By expanding cervical cancer stem cell subsets, EMT can also induce chemotherapy and radiotherapy resistance [40]. Therefore, inhibiting EMT-related mesenchymal molecules may be an effective strategy to eliminate EMT-triggering effects.

This paper first found that SCD1 knockdown could significantly inhibit the EMT process of cervical cancer cells. Evidence shows that tumor cells obtain new membrane structures through the de novo synthesis of excess fatty acids, which contain some special lipid components to form lipid rafts to promote the activation of cell growth-related receptors [41]. Therefore, SCD1 in cervical cancer cells may promote fatty acid synthesis and accelerate the growth of tumor. Furthermore, a previous study displayed that folic acid metabolism was associated to lipid synthesis [42], and folic acid deficiency and HPV16 infection might have a synergistic effect in the process of cervical carcinogenesis [43]. This suggests that fatty acid biosynthesis may be relevant to HPV16 infection, in other words, we suspect that SCD1 is somehow related to HPV infection. However, there is no evidence so far, and more research merits implement in the future. Thereafter the JASPAR database was used to predict that the transcription factor KLF9 may bind to the SCD1 promoter and regulate its expression. KLF9 has actually been found to play a transcriptional regulatory role in various types of human malignancies. The expression of KLF9 was found to be relatively low in pancreatic cancer tissue samples and cell lines, and KLF9 overexpression reduces pancreatic cancer cell proliferation, induces apoptosis, disrupts the S-phase cell cycle, and inhibits cell migration and invasion [44]. To our knowledge, there is currently no report on the regulatory role of KLF9 in cervical cancer. Herein, according to the GEPIA database, KLF9 is down-regulated in cervical cancer and correlated with staging.

Given that KLF9 could regulate SCD1 on the basis of our experimental results, we further studied the pathway of action. The Akt signaling pathway is the primary regulator of GSK3β activation, and Akt can phosphorylate GSK3β to promote the ubiquitination and degradation of GSK3β [45]. Phosphorylated GSK3β also increases SNAIL phosphorylation and nuclear export, activates, and promotes EMT, thus, the Akt/GSK3β pathway can affect EMT, which in turn affects tumor aggressiveness [46]. The experimental results in this paper indicate that KLF9/SCD1 expression can regulate the Akt/GSK3β signaling pathway in cervical cancer cells. De novo synthesis of fatty acids in tumor cells contributes to the production of lipids involved in regulating the activity of proto-oncogenes, such as phosphatidylinositol, phosphatidylserine, and lecithin. They are important factors in activating proliferation- and survival-related signaling pathways, especially in AKT, Ras and Wnt signaling pathways [47]. These studies have indicated that tumor cells synthesize excess lipids, on the one hand, promote the formation of cell membranes and support rapid division, and on the other hand, use lipid metabolism intermediates or post-translational modifications to positively regulate pro-proliferation and survival-related pathways.

The finding of the KLF9/SCD1/Akt/GSK3β regulatory axis expands the pathogenesis of cervical cancer. Nevertheless, this paper is limited to *in vitro* experiments, and more *in vivo* studies are required to verify. Together, the present study indicates that SCD1 is negatively regulated by KLF9 and it activates the Akt/GSK3β signaling pathway to influence the proliferation, migration, invasion, and EMT process of cervical cancer cells. The present study provides a novel target for the development of prevention and drugs.

## Conclusion

Due to the heterogeneity of tumor cells [48], targeted therapy that inhibits individual cancer-promoting signaling pathways is ineffective. Tumors of different origins have similar characteristics at the metabolic level relative to genetic mutations [49]. Therefore, tumor drug development and treatment targeting tumor metabolic characteristics have unique advantages. In-depth understanding of the specific mechanism of fatty acid synthesis-related genes in tumor cells, inhibition of fatty acid synthesis specific to malignant tumor cells (such as SCD1 inhibitors), has provided novel ideas for the biological treatment of malignant tumors.

## Supplementary Material

Supplemental Material

## Data Availability

The datasets are available from the corresponding author on reasonable request.
