# Supplementary figures and images for: Stearoyl-CoA desaturase 1 regulates malignant progression of cervical cancer cells
Source: Bioengineered. 2022 May 24;13(5):12941–54. doi: 10.1080/21655979.2022.2079253 (PMC9275951; doi:10.1080/21655979.2022.2079253)

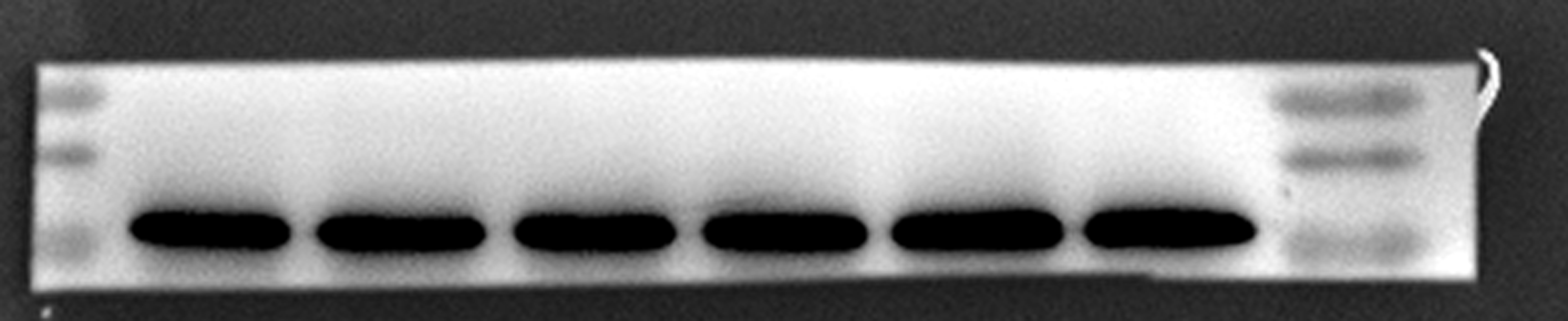

Supplement: Supplemental Material [file KBIE_A_2079253_SM0231.zip › blots/Fig1/gapdh.tif]

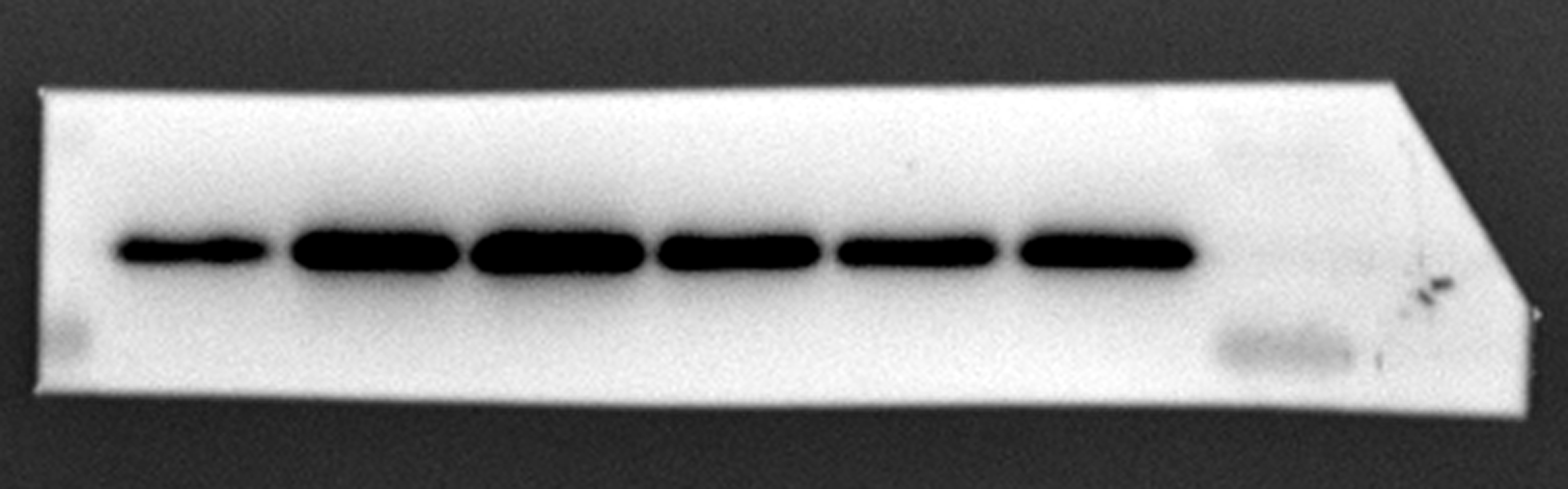

Supplement: Supplemental Material [file KBIE_A_2079253_SM0231.zip › blots/Fig1/SCD1.tif]

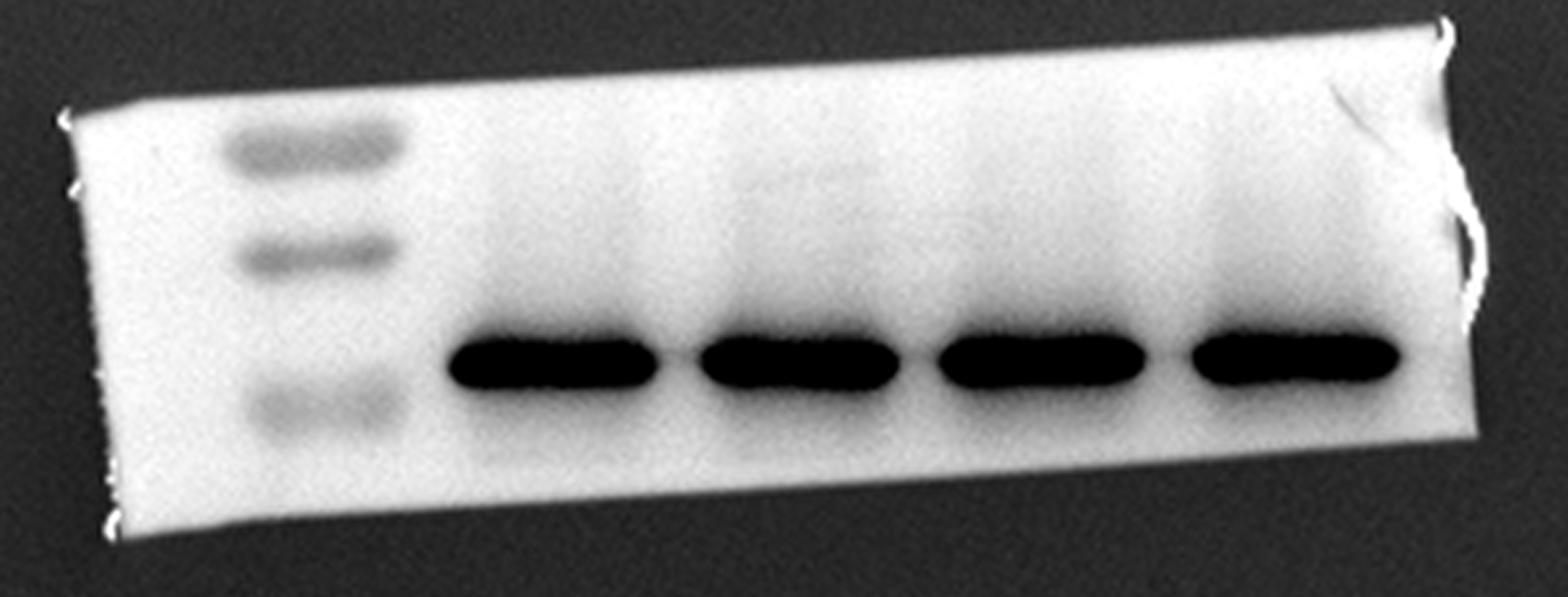

Supplement: Supplemental Material [file KBIE_A_2079253_SM0231.zip › blots/Fig2B/gapdh.tif]

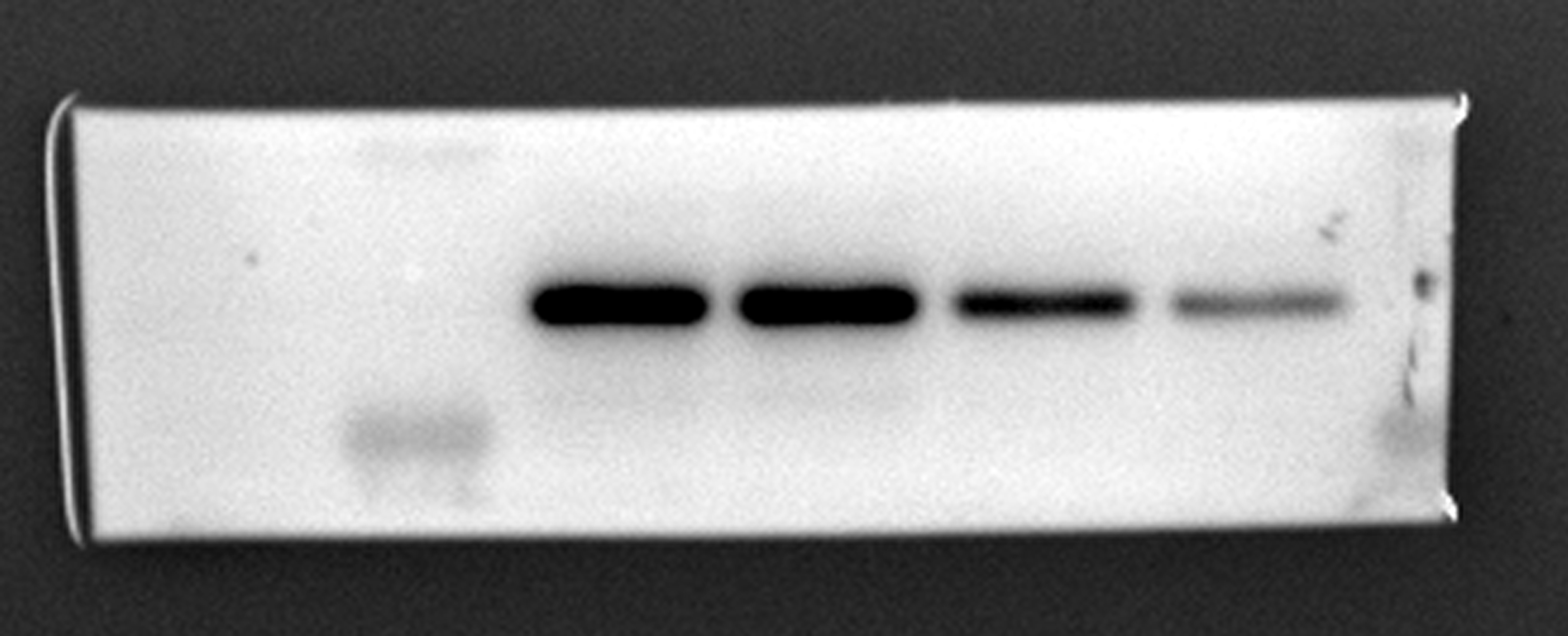

Supplement: Supplemental Material [file KBIE_A_2079253_SM0231.zip › blots/Fig2B/SCD1.tif]

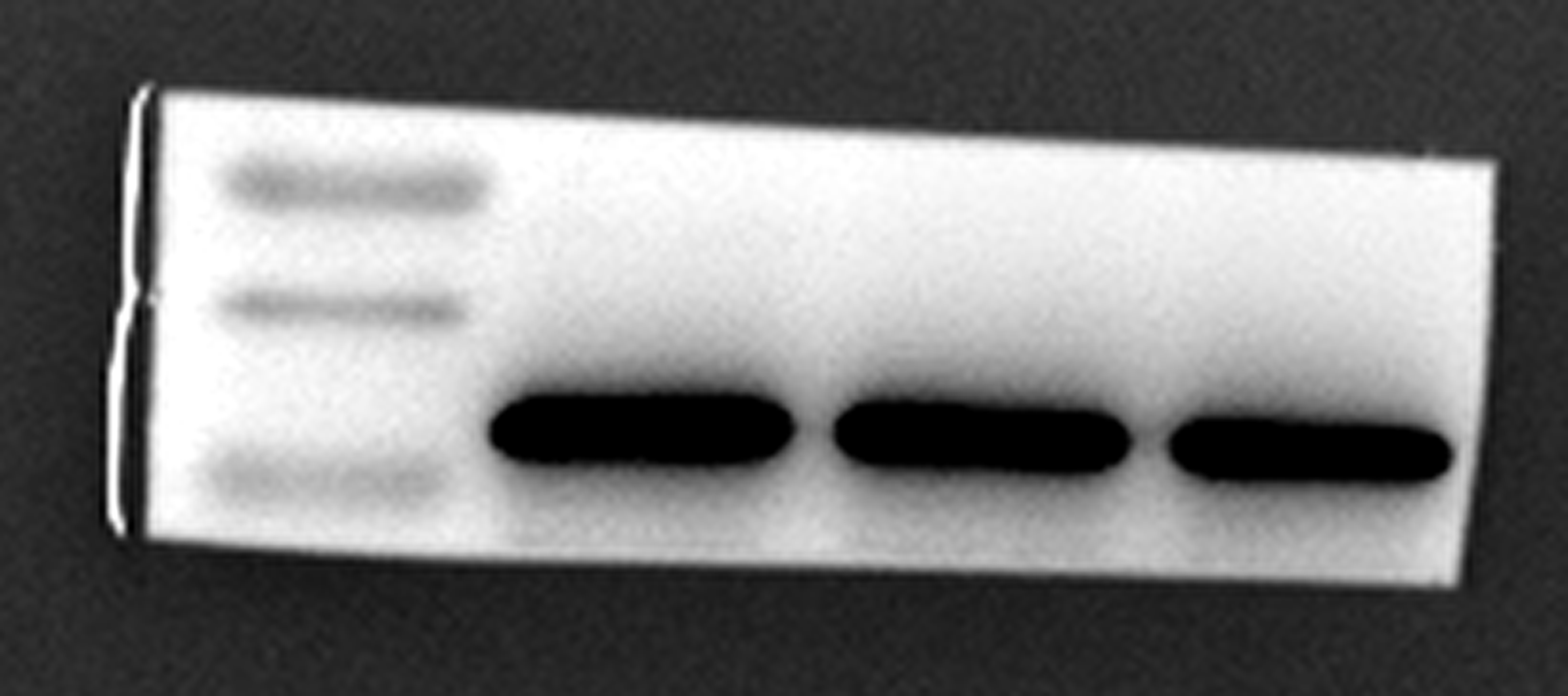

Supplement: Supplemental Material [file KBIE_A_2079253_SM0231.zip › blots/Fig2E/gapdh.tif]

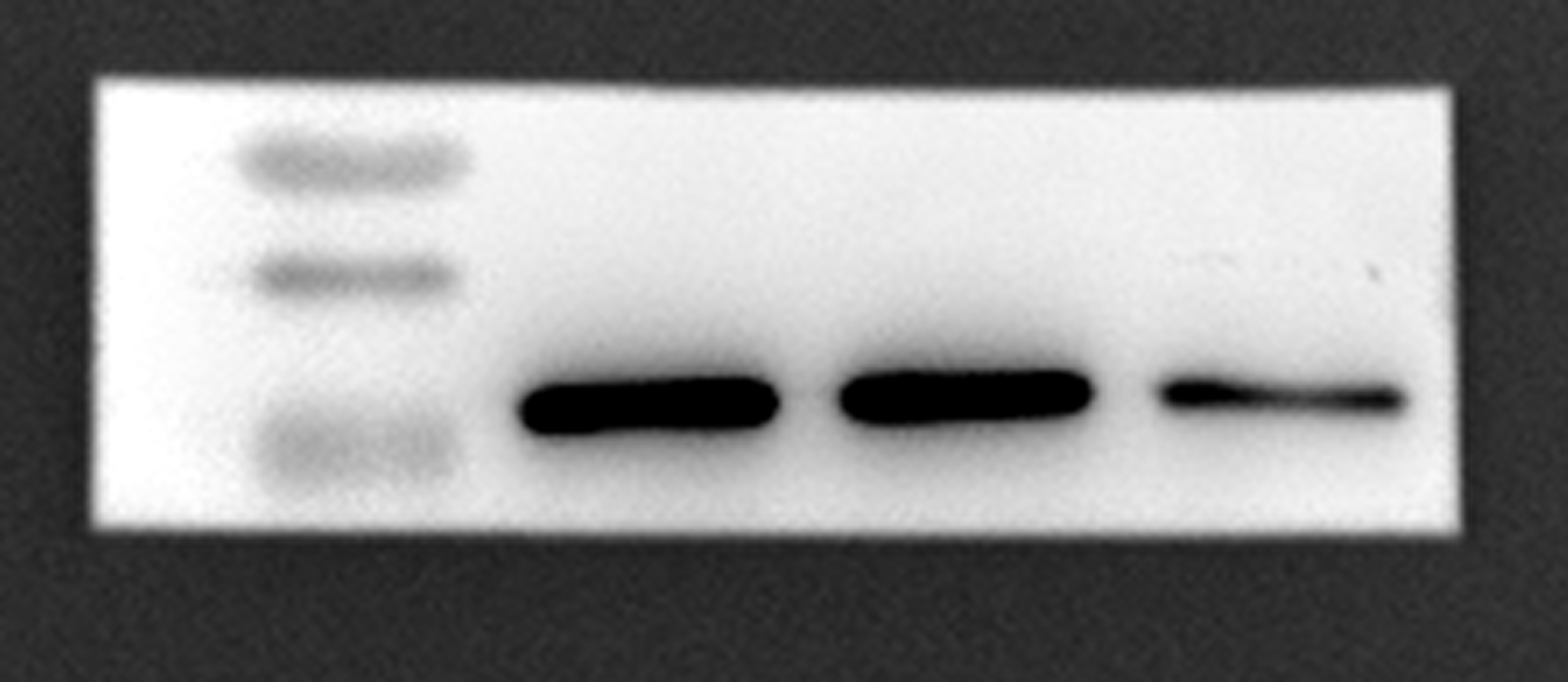

Supplement: Supplemental Material [file KBIE_A_2079253_SM0231.zip › blots/Fig2E/ki67.tif]

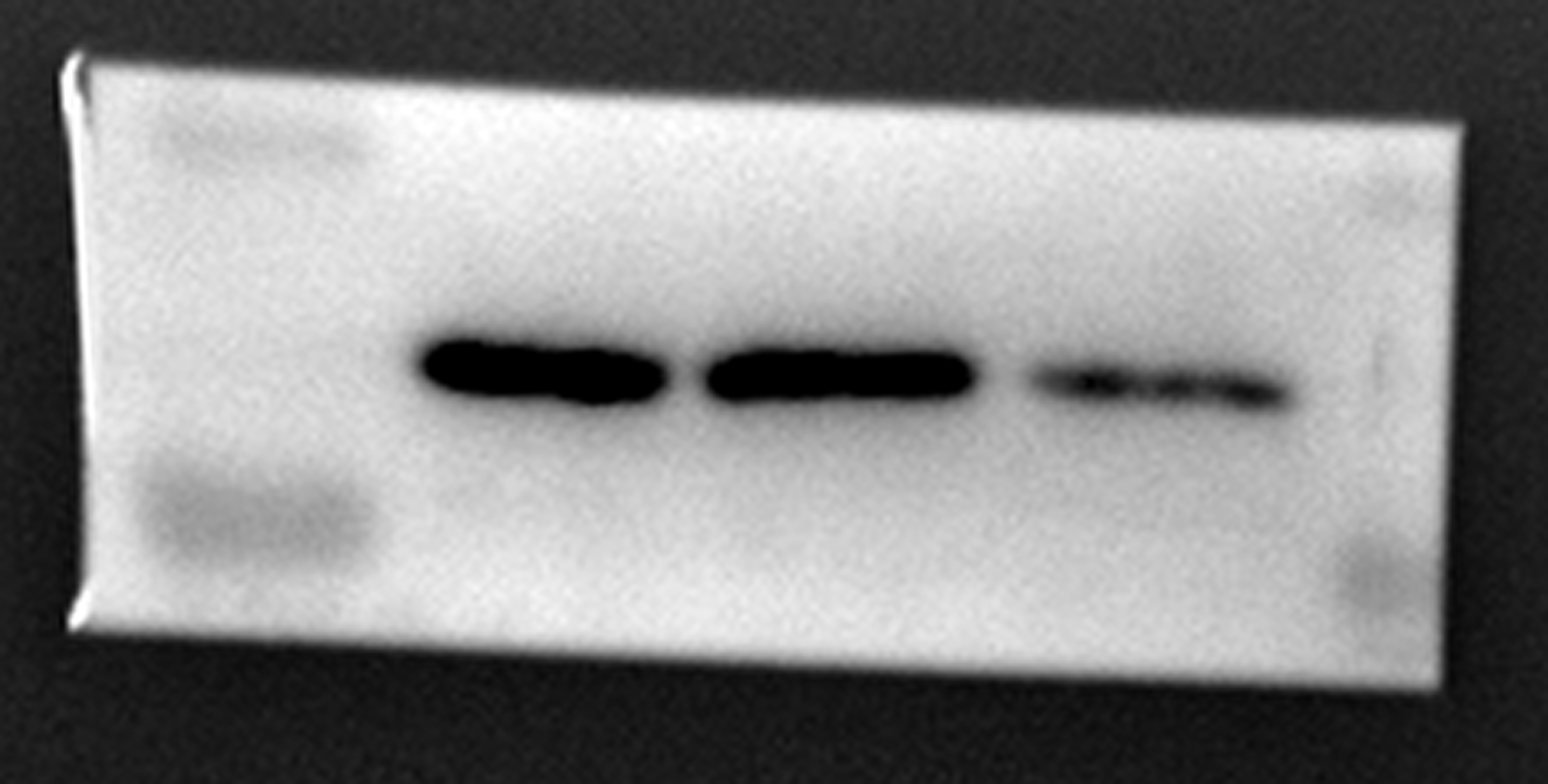

Supplement: Supplemental Material [file KBIE_A_2079253_SM0231.zip › blots/Fig2E/PCNA.tif]

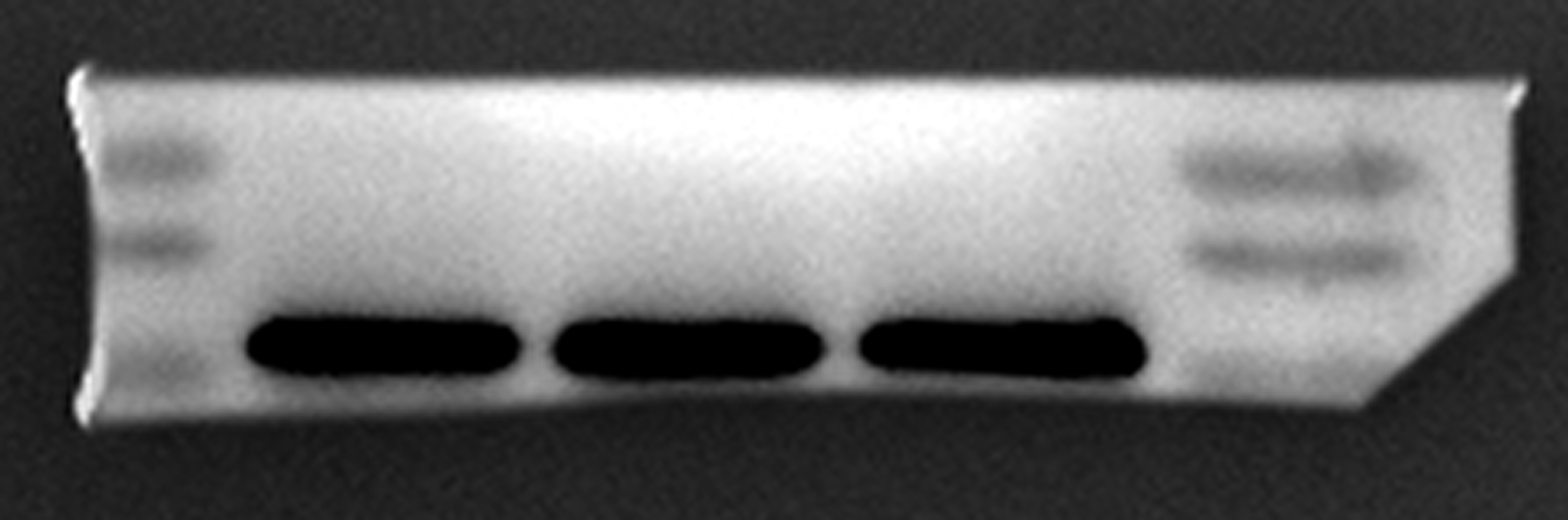

Supplement: Supplemental Material [file KBIE_A_2079253_SM0231.zip › blots/Fig3C/gapdh-1.tif]

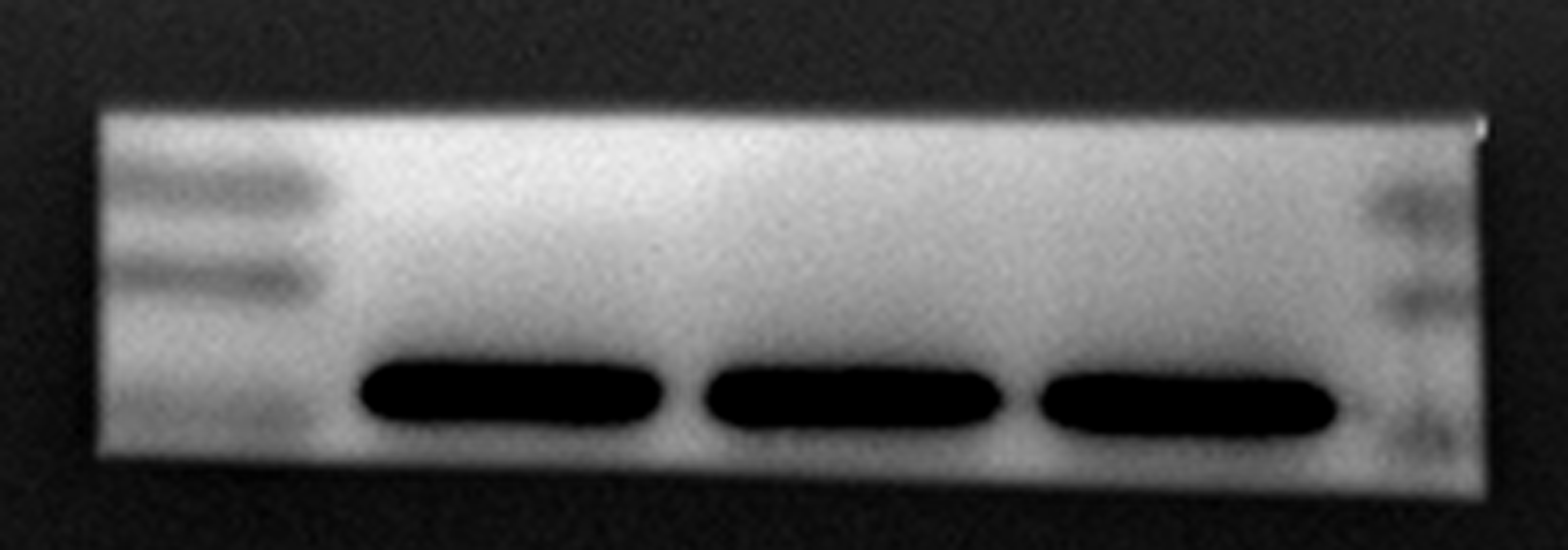

Supplement: Supplemental Material [file KBIE_A_2079253_SM0231.zip › blots/Fig3C/gapdh.tif]

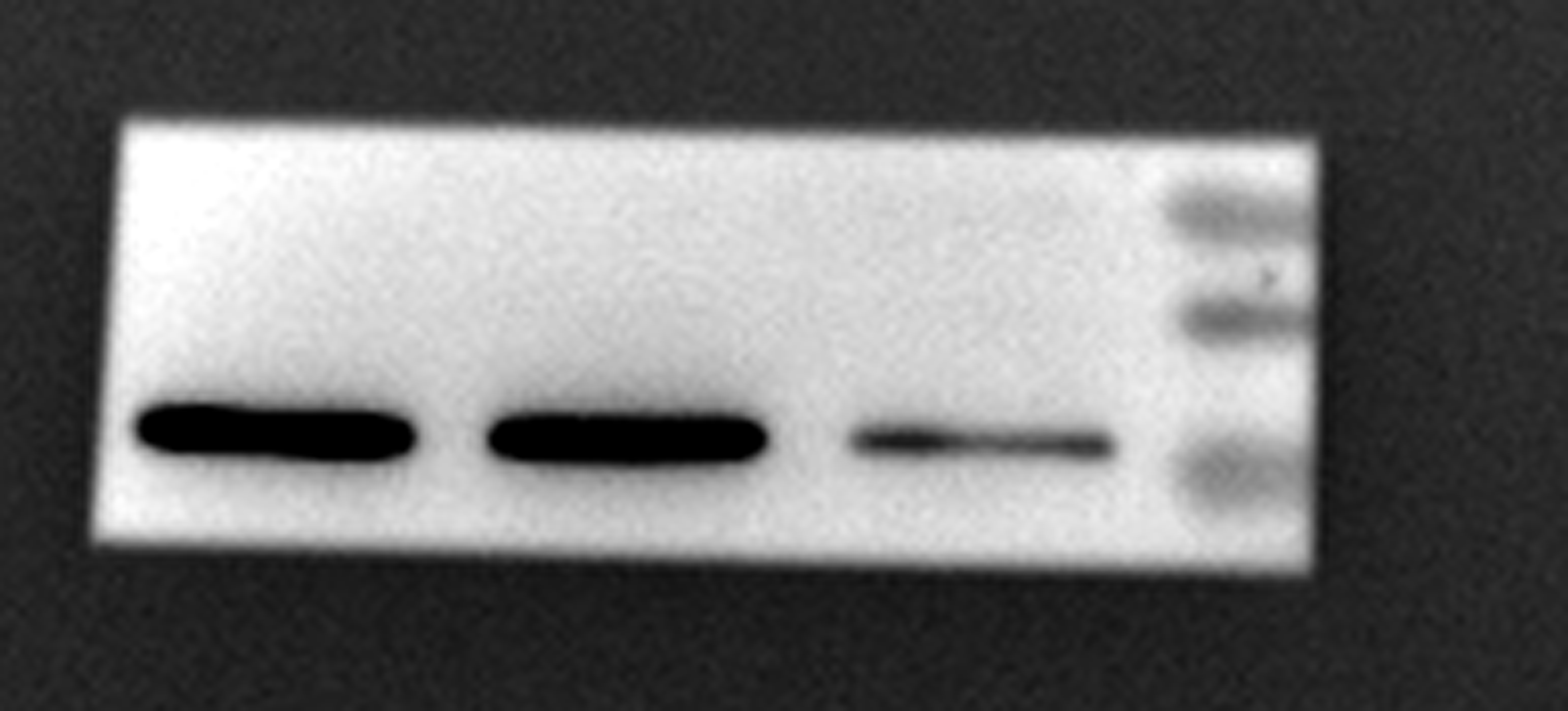

Supplement: Supplemental Material [file KBIE_A_2079253_SM0231.zip › blots/Fig3C/MMP2.tif]

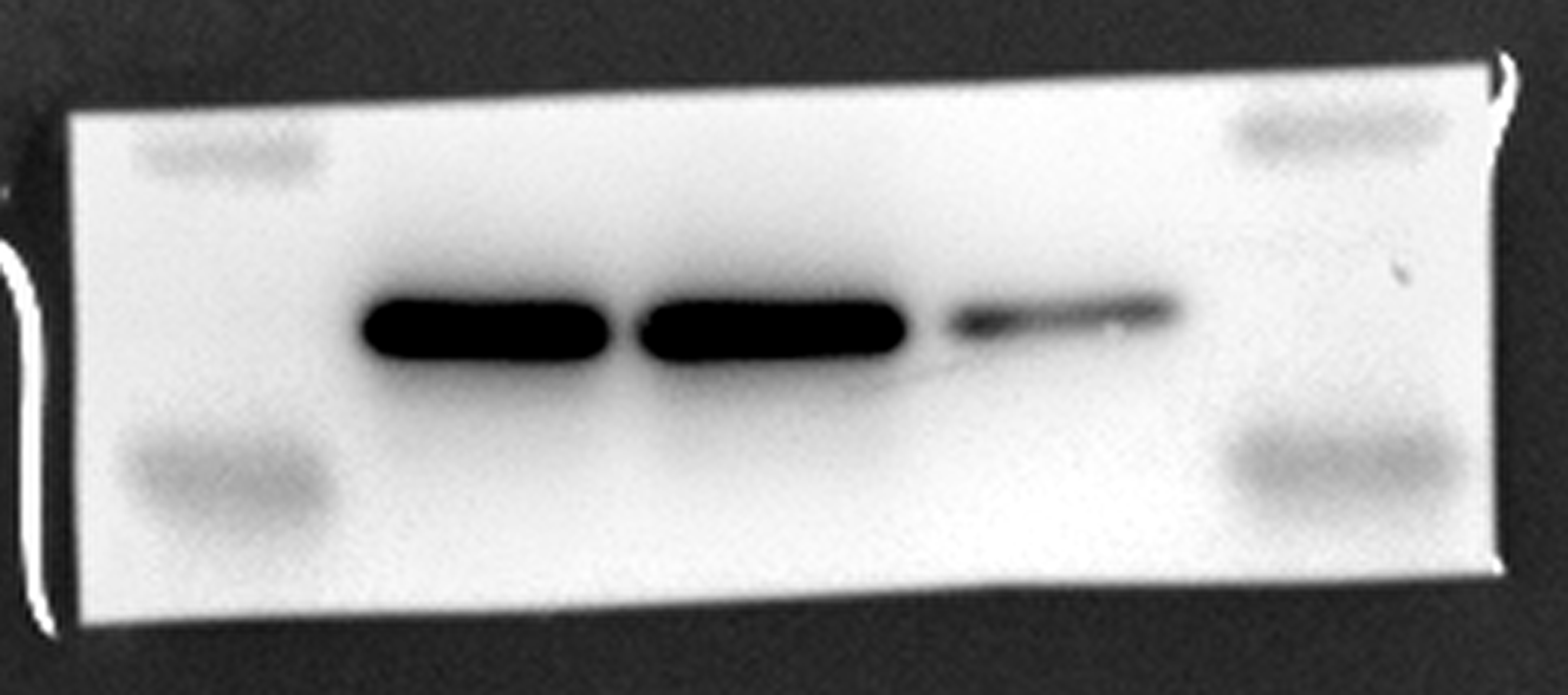

Supplement: Supplemental Material [file KBIE_A_2079253_SM0231.zip › blots/Fig3C/MMP9.tif]

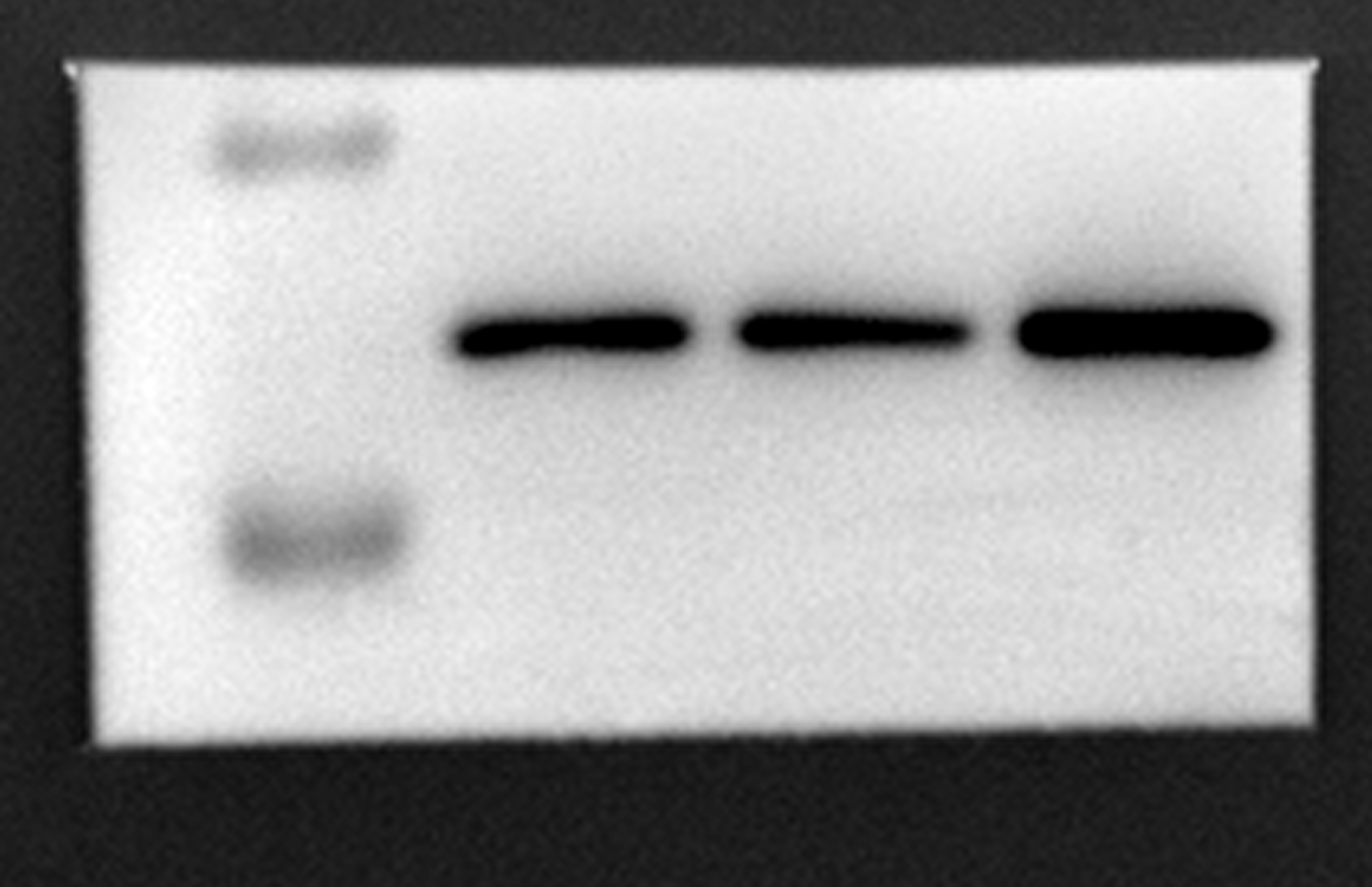

Supplement: Supplemental Material [file KBIE_A_2079253_SM0231.zip › blots/Fig3D/E-cadherin.tif]

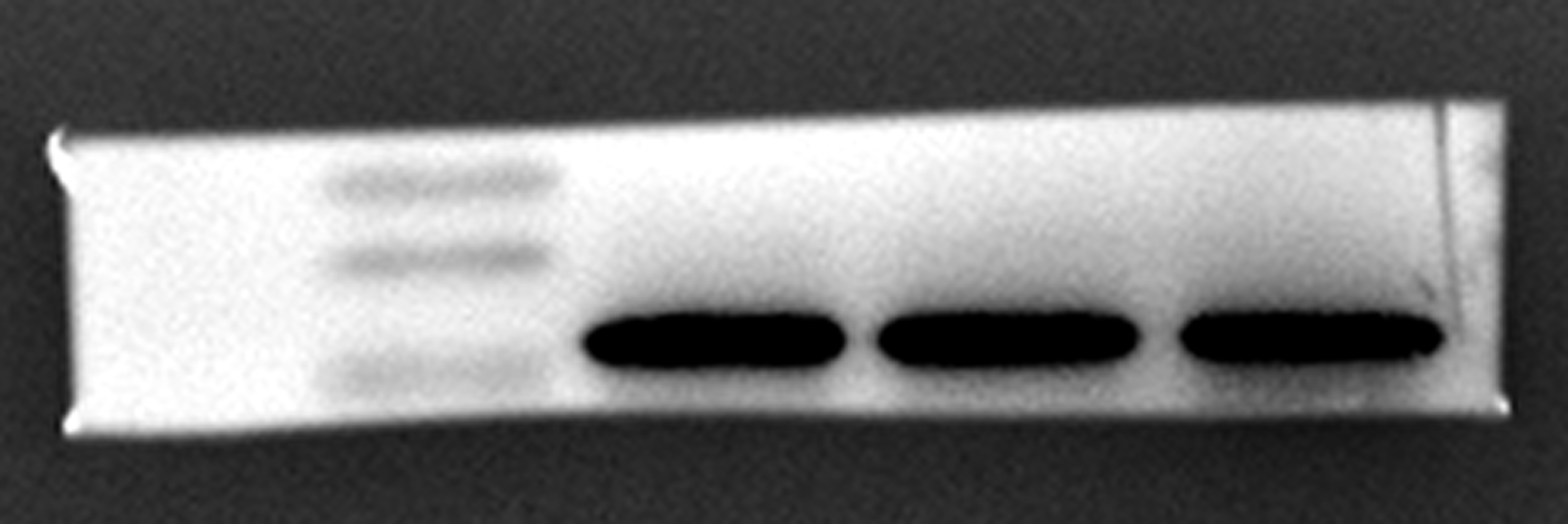

Supplement: Supplemental Material [file KBIE_A_2079253_SM0231.zip › blots/Fig3D/gapdh-1.tif]

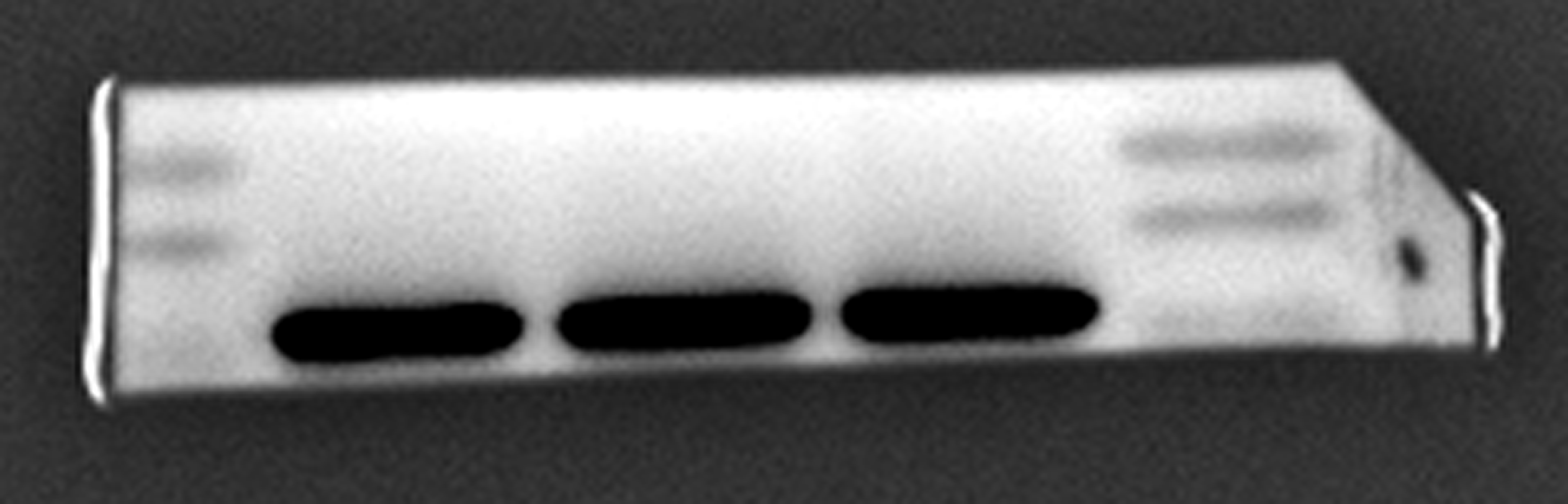

Supplement: Supplemental Material [file KBIE_A_2079253_SM0231.zip › blots/Fig3D/gapdh.tif]

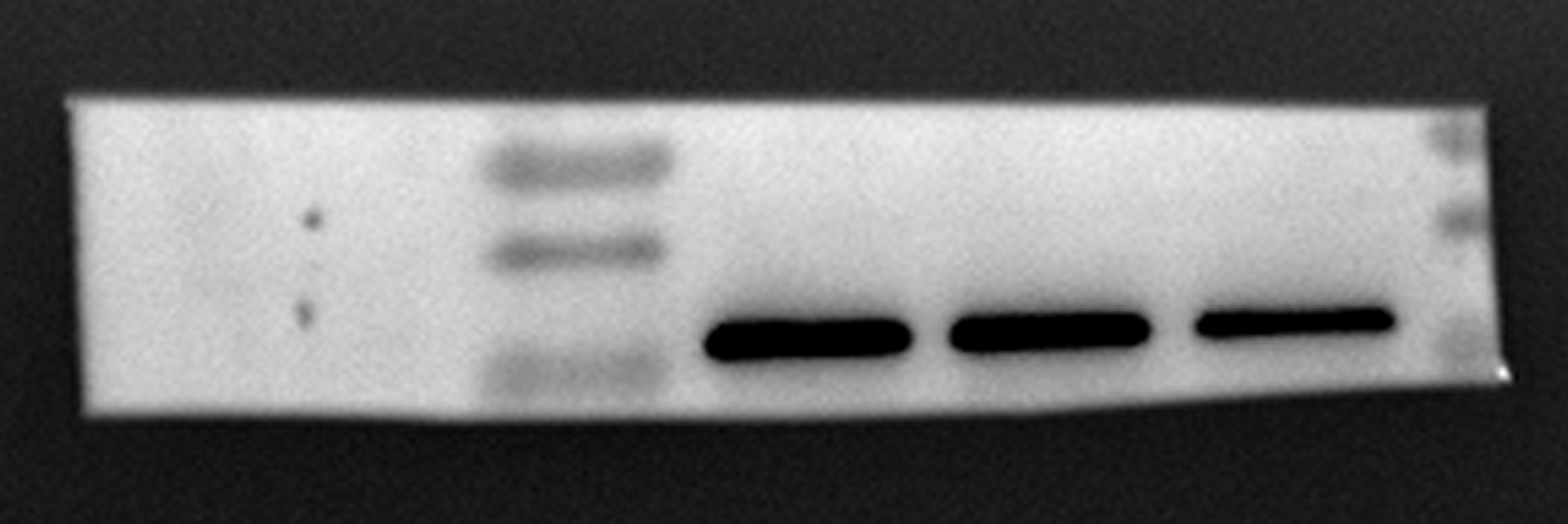

Supplement: Supplemental Material [file KBIE_A_2079253_SM0231.zip › blots/Fig3D/N-cadherin.tif]

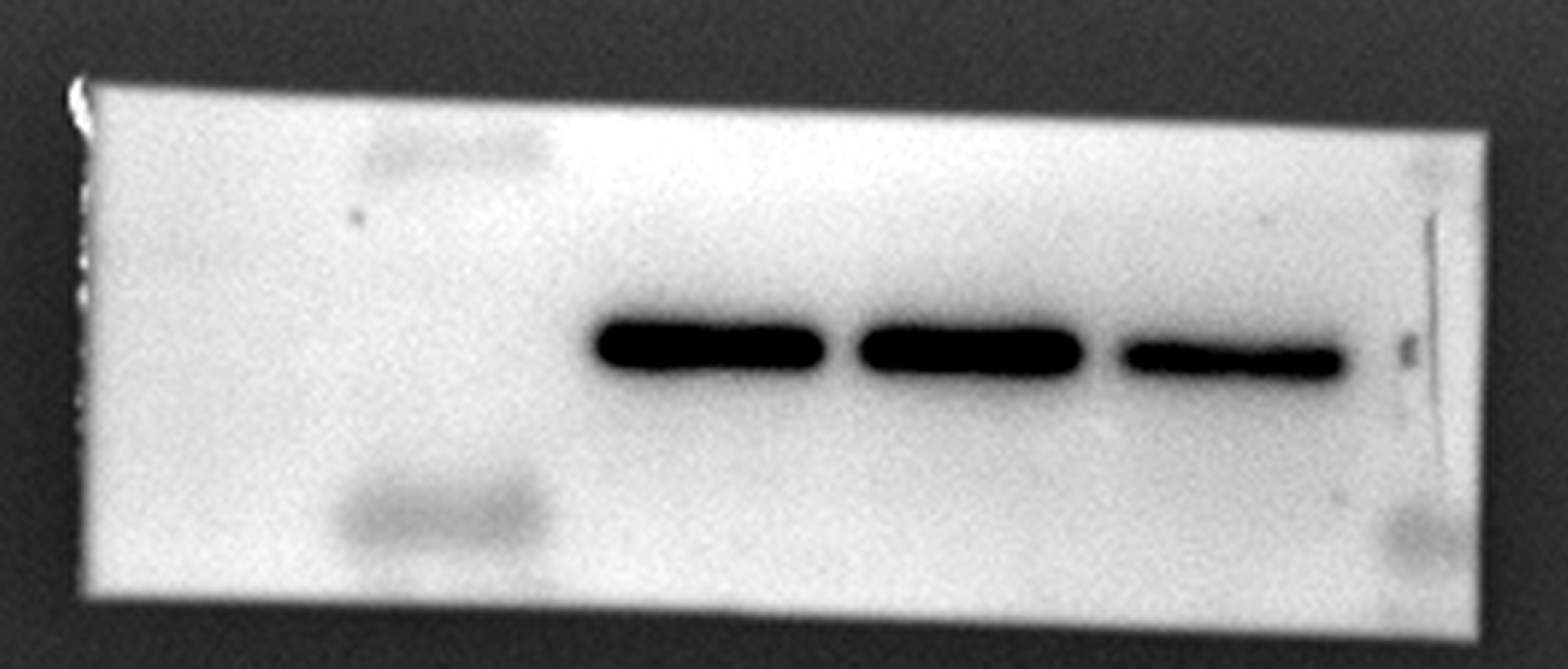

Supplement: Supplemental Material [file KBIE_A_2079253_SM0231.zip › blots/Fig3D/Snail.tif]

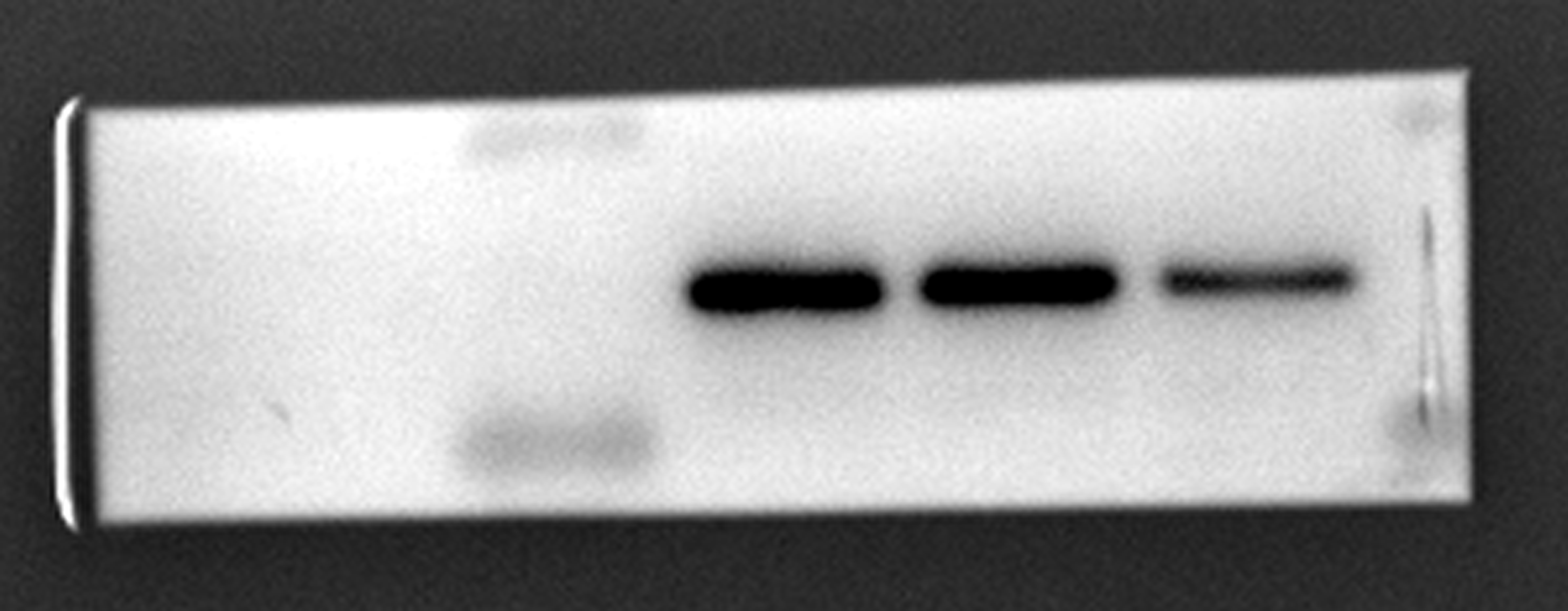

Supplement: Supplemental Material [file KBIE_A_2079253_SM0231.zip › blots/Fig3D/vimentin.tif]

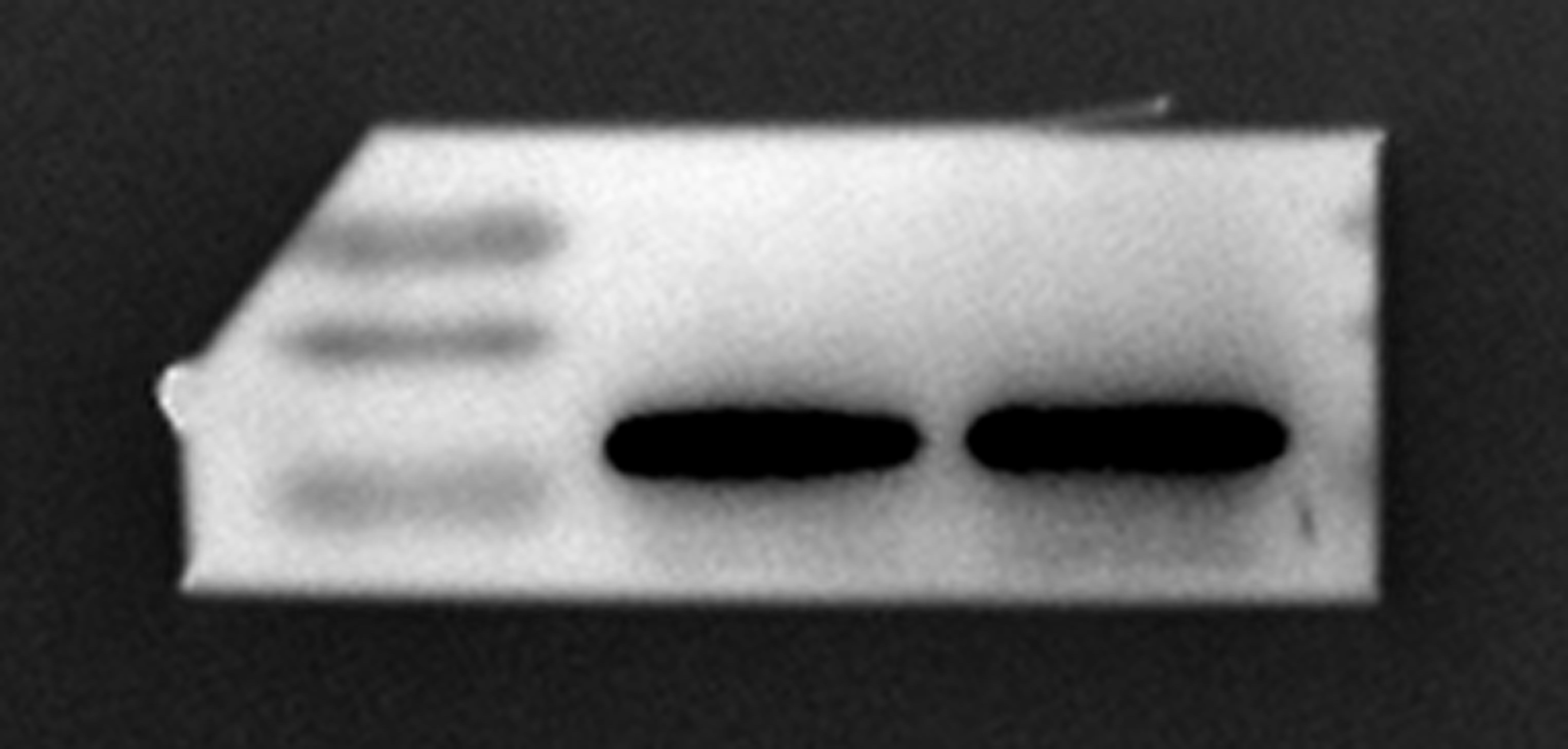

Supplement: Supplemental Material [file KBIE_A_2079253_SM0231.zip › blots/Fig4E/gapdh.tif]

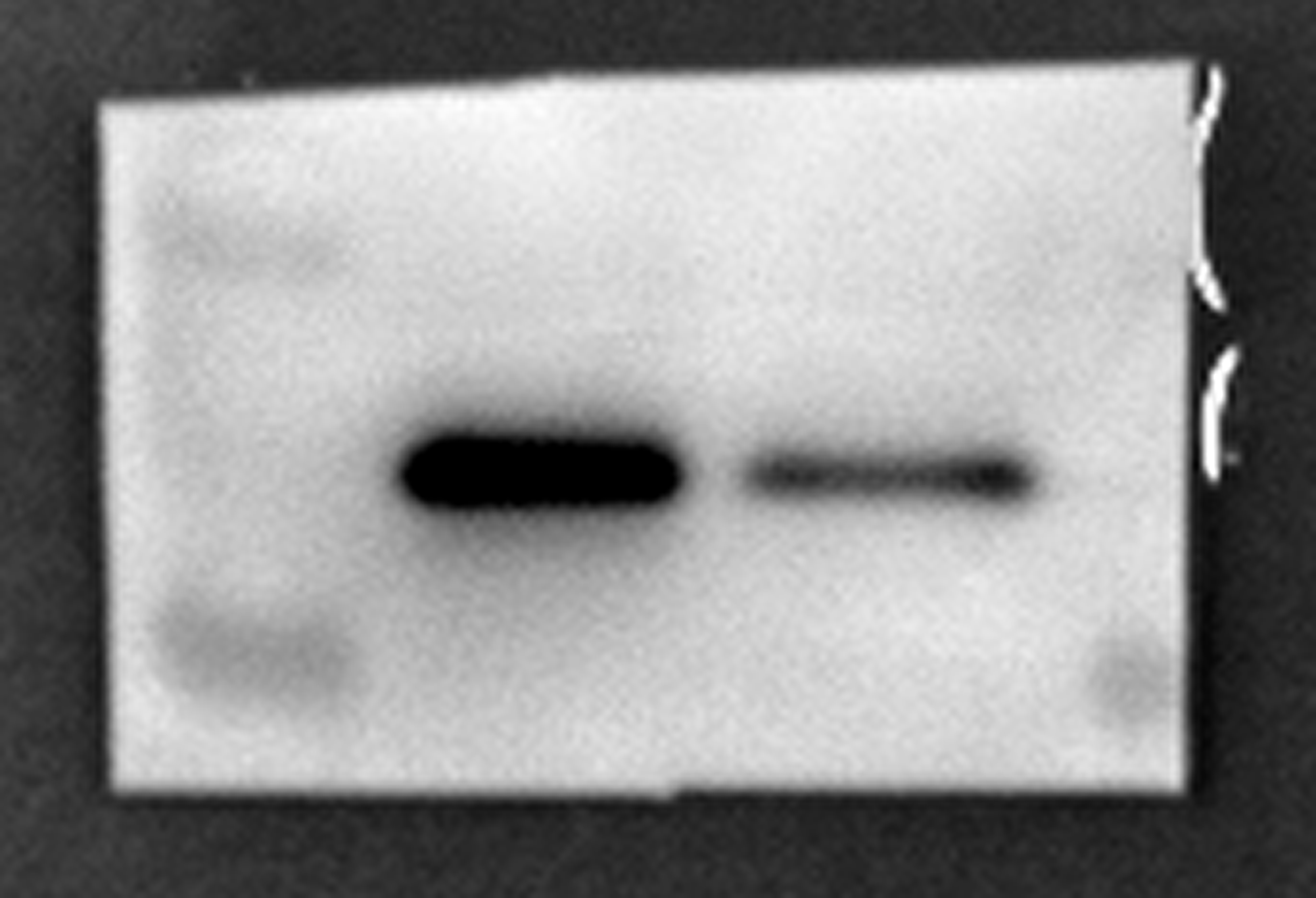

Supplement: Supplemental Material [file KBIE_A_2079253_SM0231.zip › blots/Fig4E/KLF9.tif]

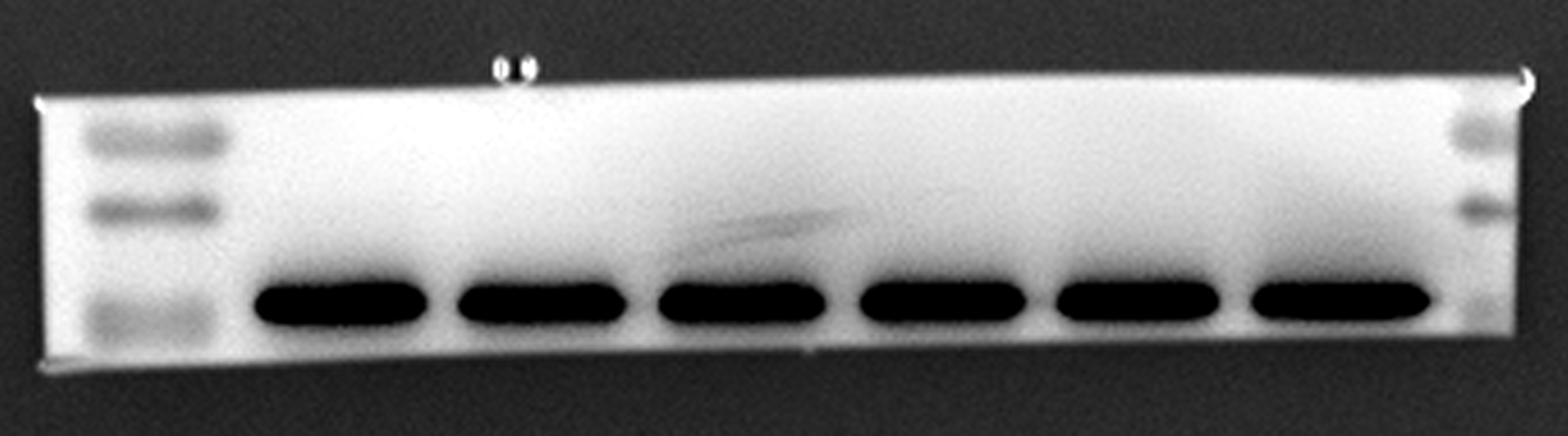

Supplement: Supplemental Material [file KBIE_A_2079253_SM0231.zip › blots/Fig5C/gapdh.tif]

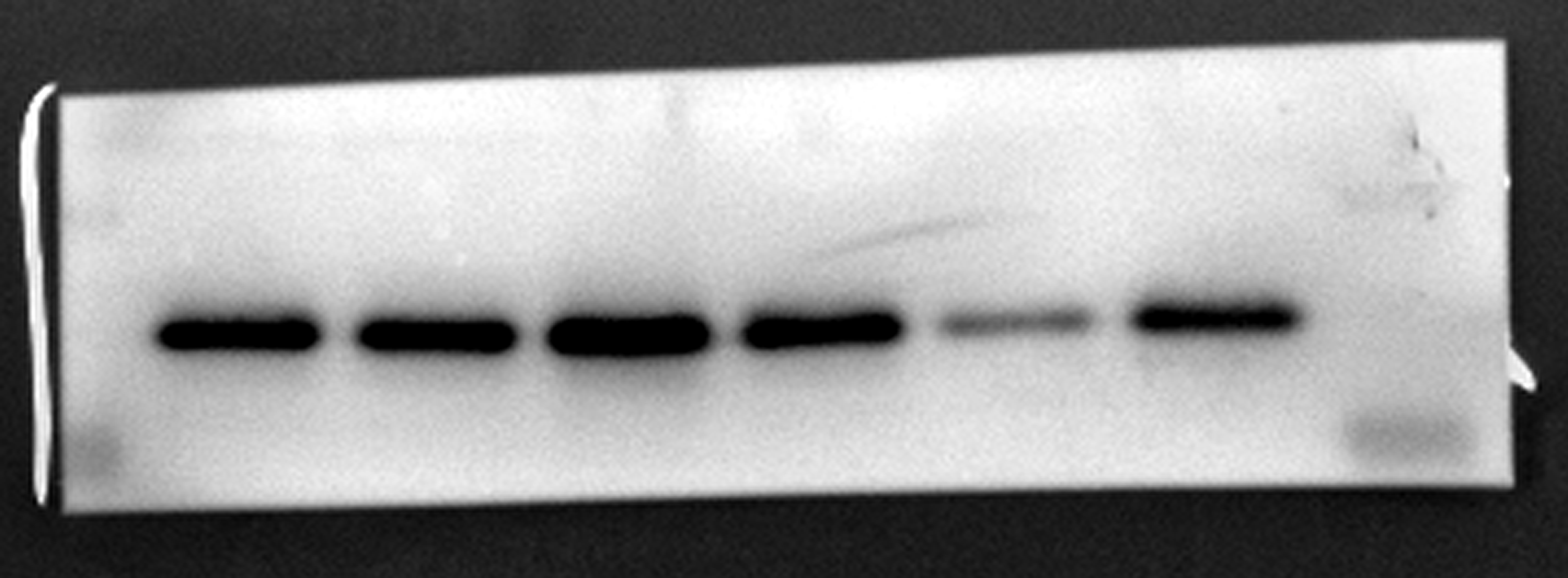

Supplement: Supplemental Material [file KBIE_A_2079253_SM0231.zip › blots/Fig5C/KLF9.tif]

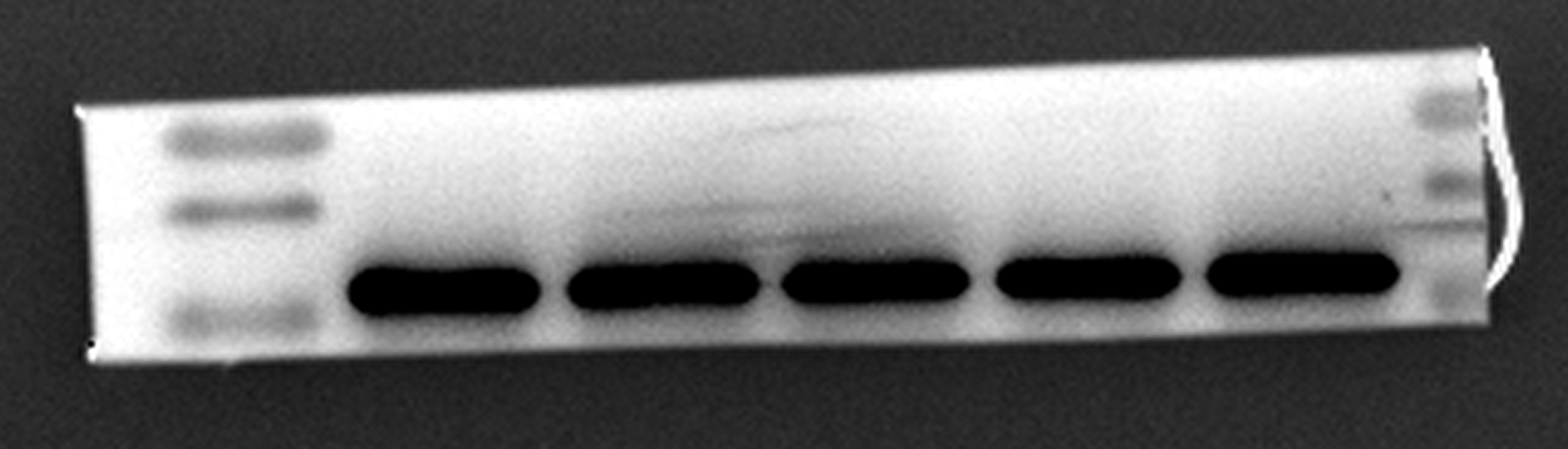

Supplement: Supplemental Material [file KBIE_A_2079253_SM0231.zip › blots/Fig5E/gapdh.tif]

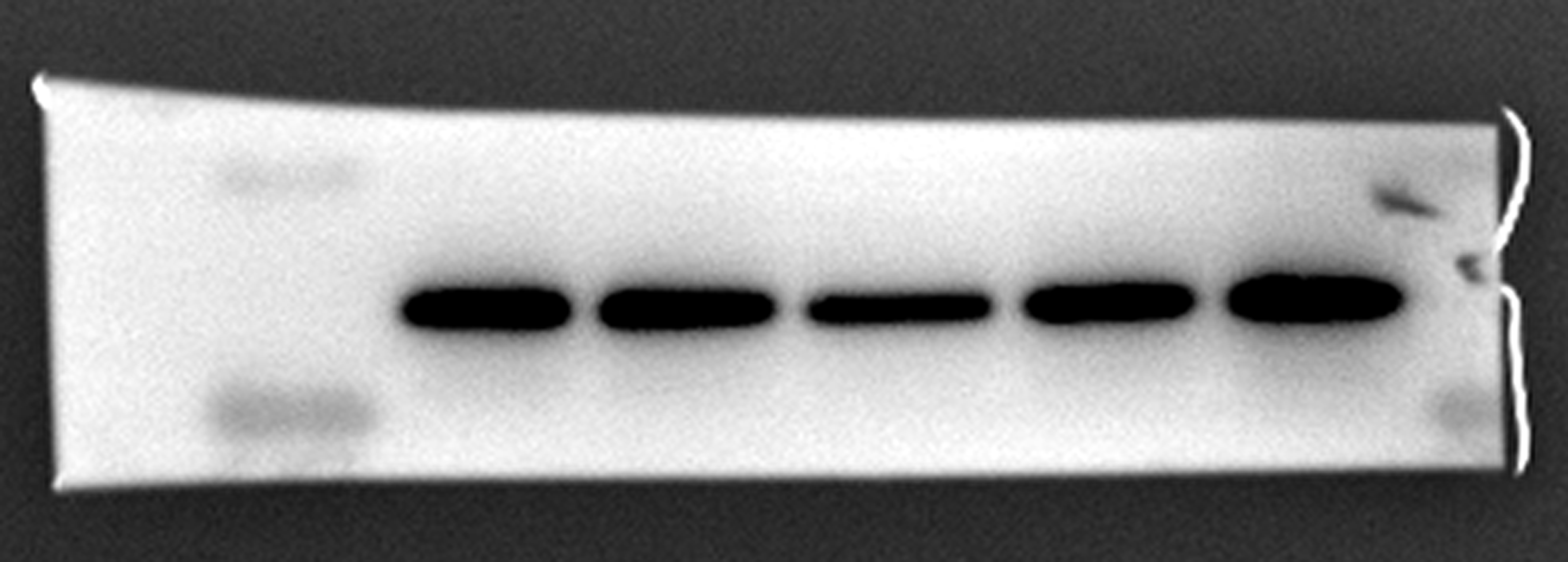

Supplement: Supplemental Material [file KBIE_A_2079253_SM0231.zip › blots/Fig5E/SCD1.tif]

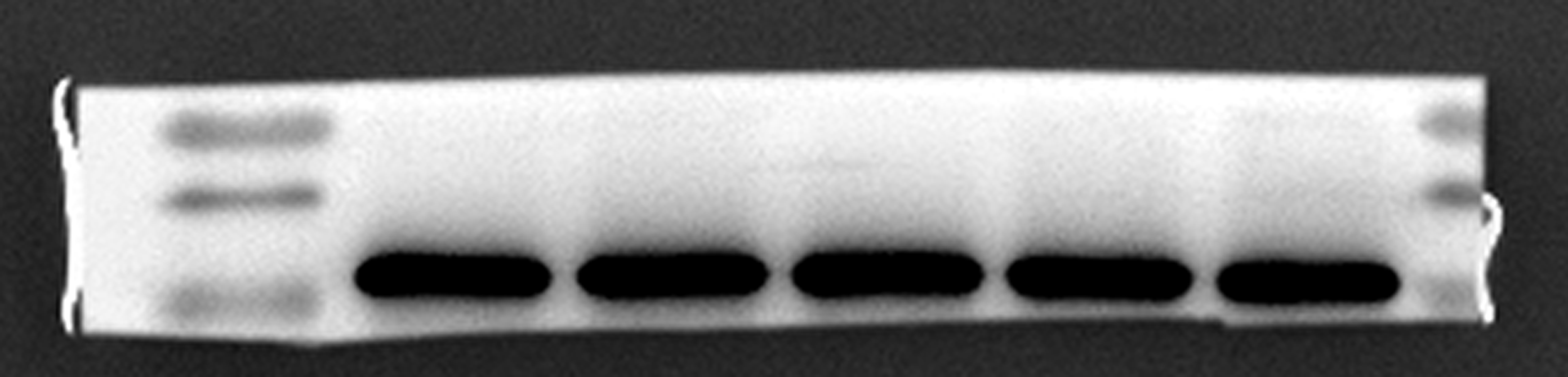

Supplement: Supplemental Material [file KBIE_A_2079253_SM0231.zip › blots/Fig6C/gapdh.tif]

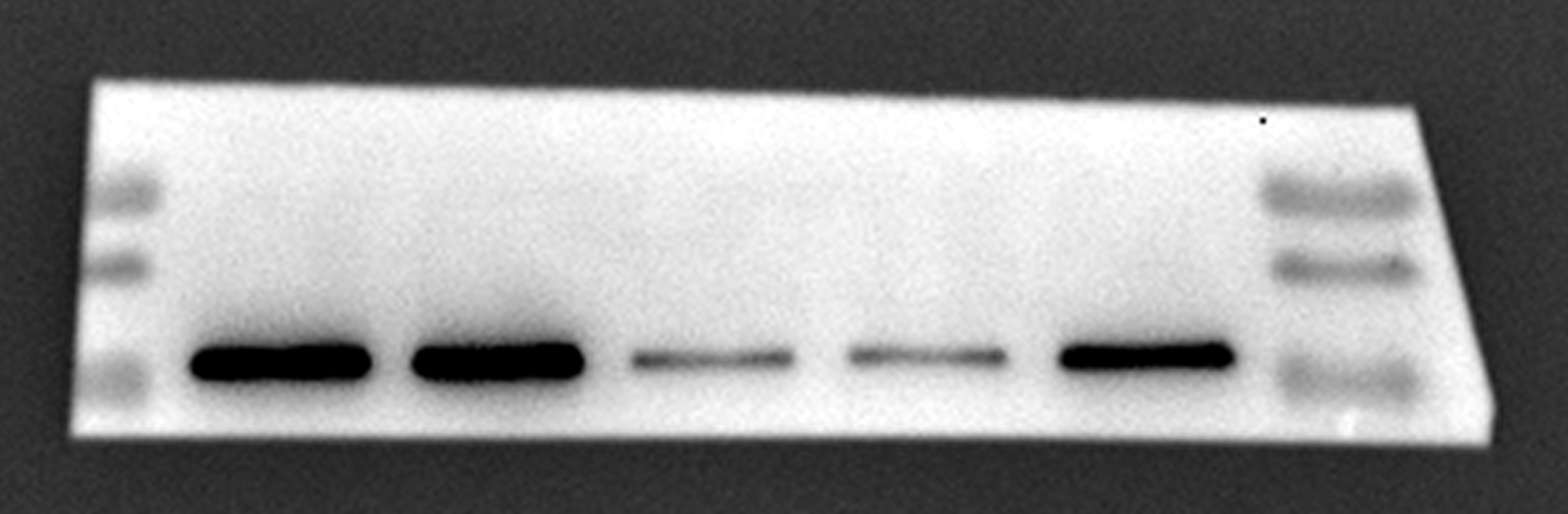

Supplement: Supplemental Material [file KBIE_A_2079253_SM0231.zip › blots/Fig6C/ki67.tif]

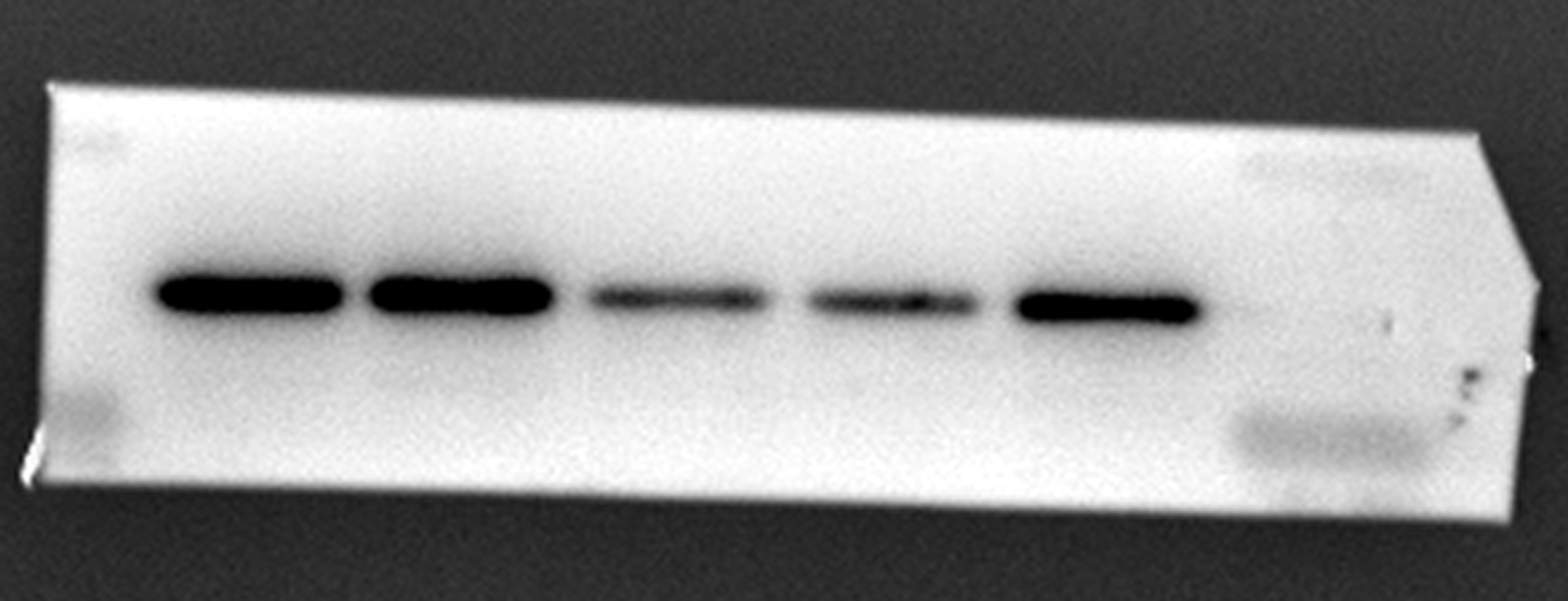

Supplement: Supplemental Material [file KBIE_A_2079253_SM0231.zip › blots/Fig6C/PCNA.tif]

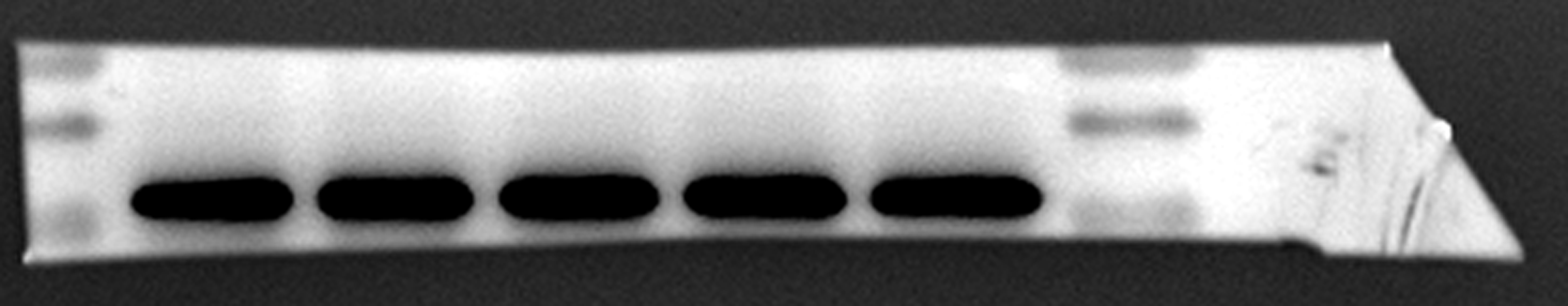

Supplement: Supplemental Material [file KBIE_A_2079253_SM0231.zip › blots/Fig7C/gapdh-1.tif]

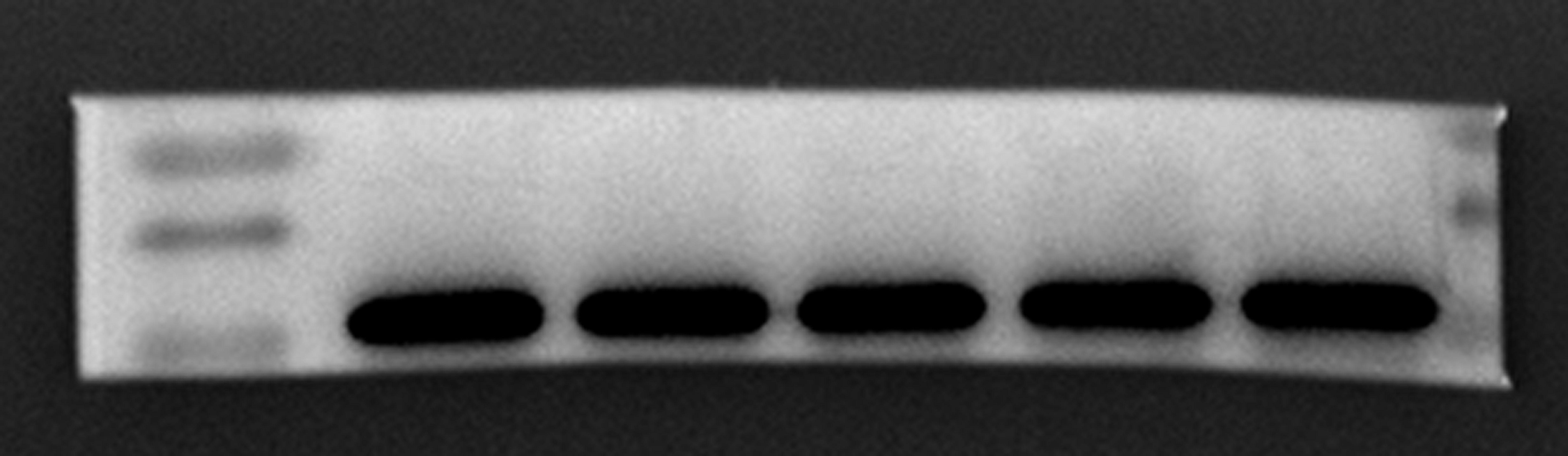

Supplement: Supplemental Material [file KBIE_A_2079253_SM0231.zip › blots/Fig7C/gapdh.tif]

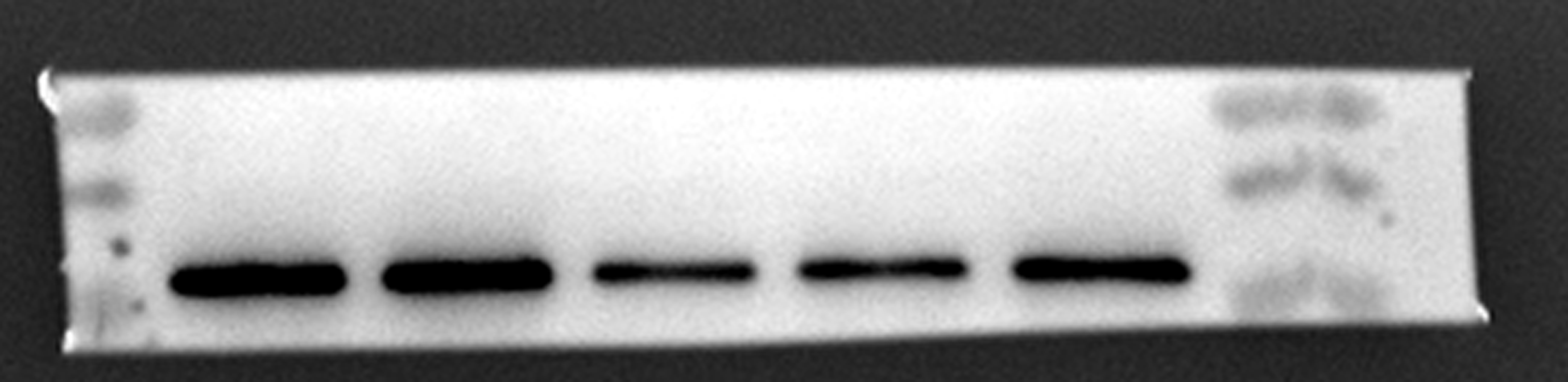

Supplement: Supplemental Material [file KBIE_A_2079253_SM0231.zip › blots/Fig7C/MMP2.tif]

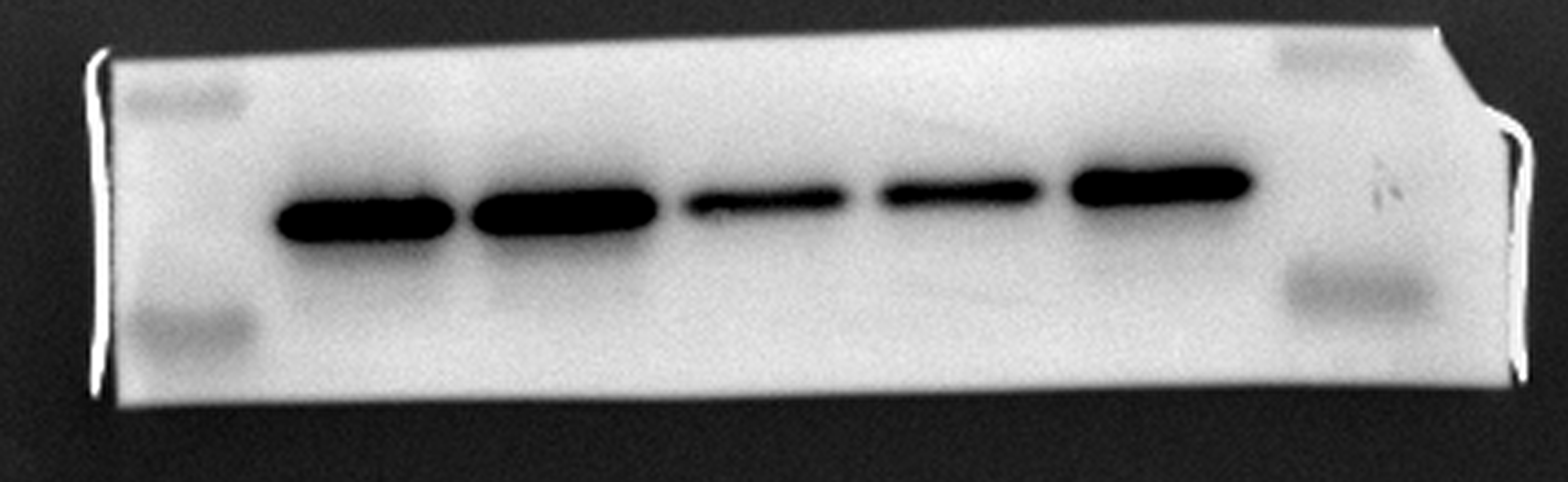

Supplement: Supplemental Material [file KBIE_A_2079253_SM0231.zip › blots/Fig7C/MMP9.tif]

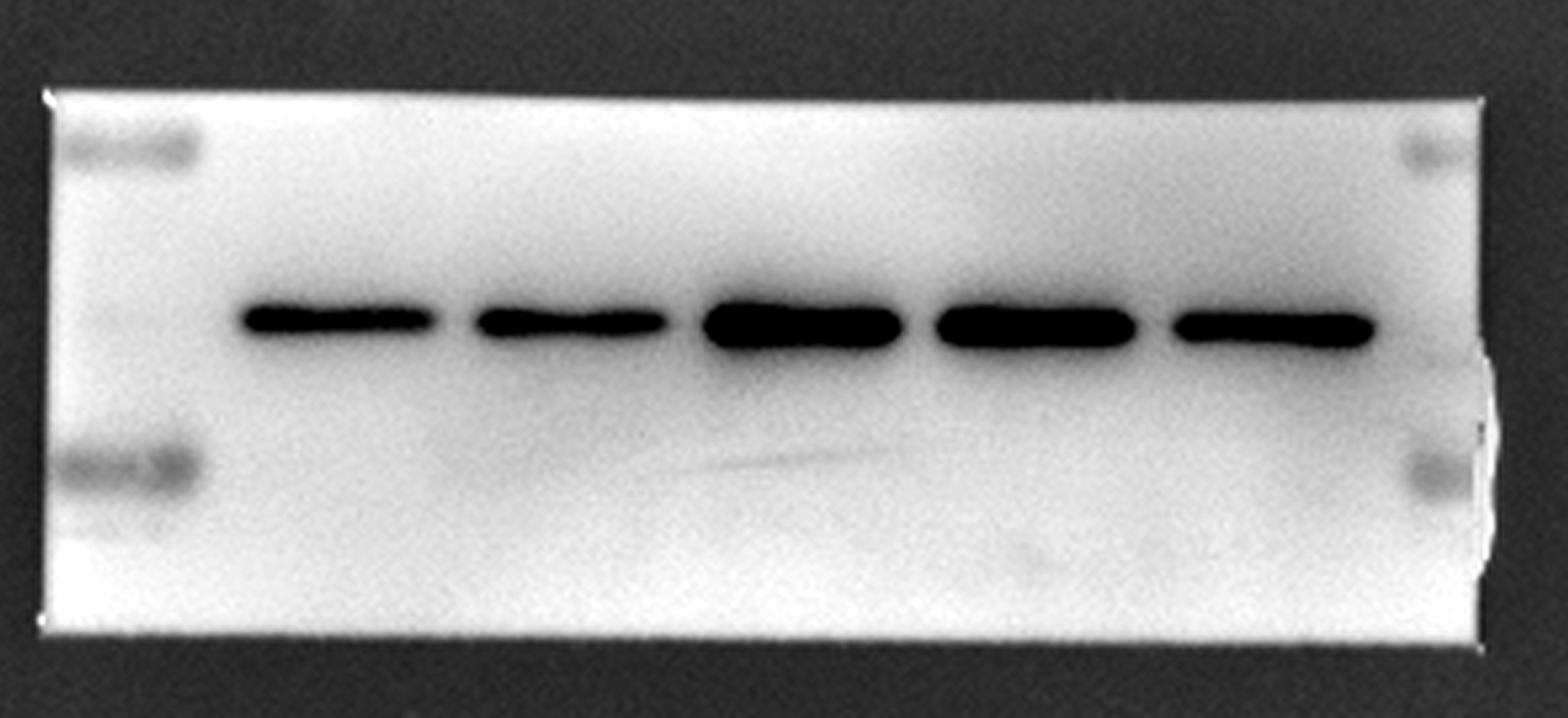

Supplement: Supplemental Material [file KBIE_A_2079253_SM0231.zip › blots/Fig7D/E-cadherin.tif]

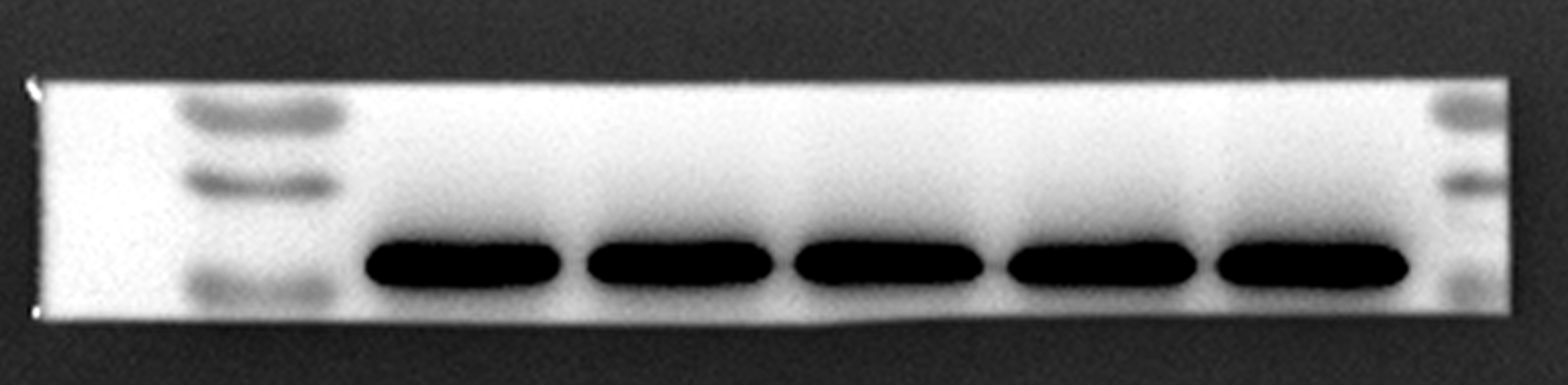

Supplement: Supplemental Material [file KBIE_A_2079253_SM0231.zip › blots/Fig7D/gapdh-1.tif]

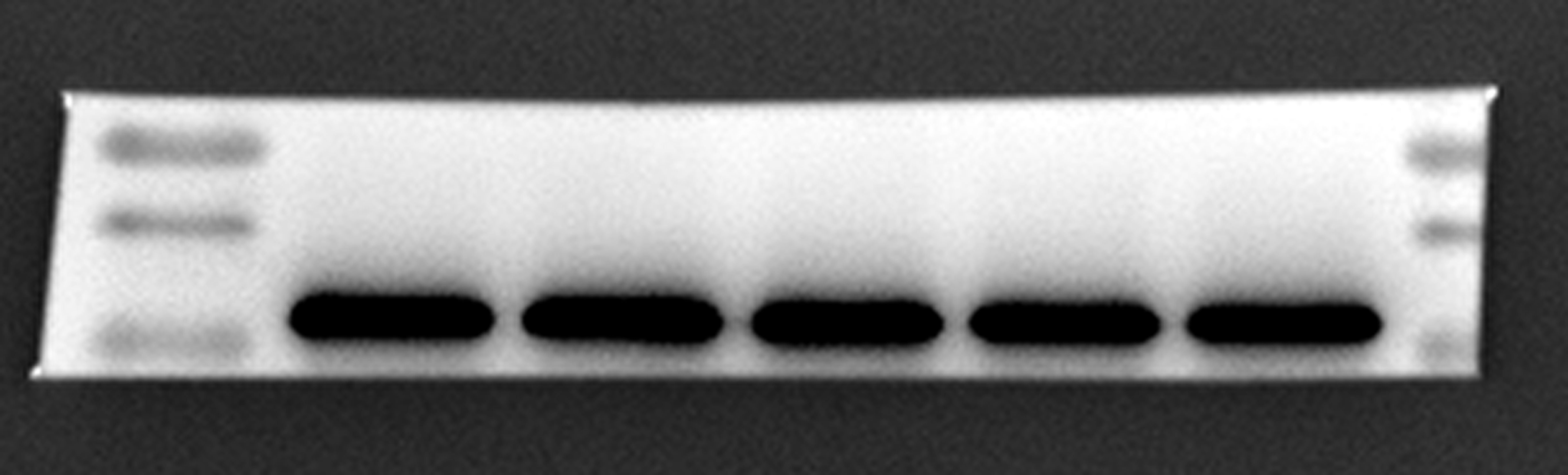

Supplement: Supplemental Material [file KBIE_A_2079253_SM0231.zip › blots/Fig7D/gapdh.tif]

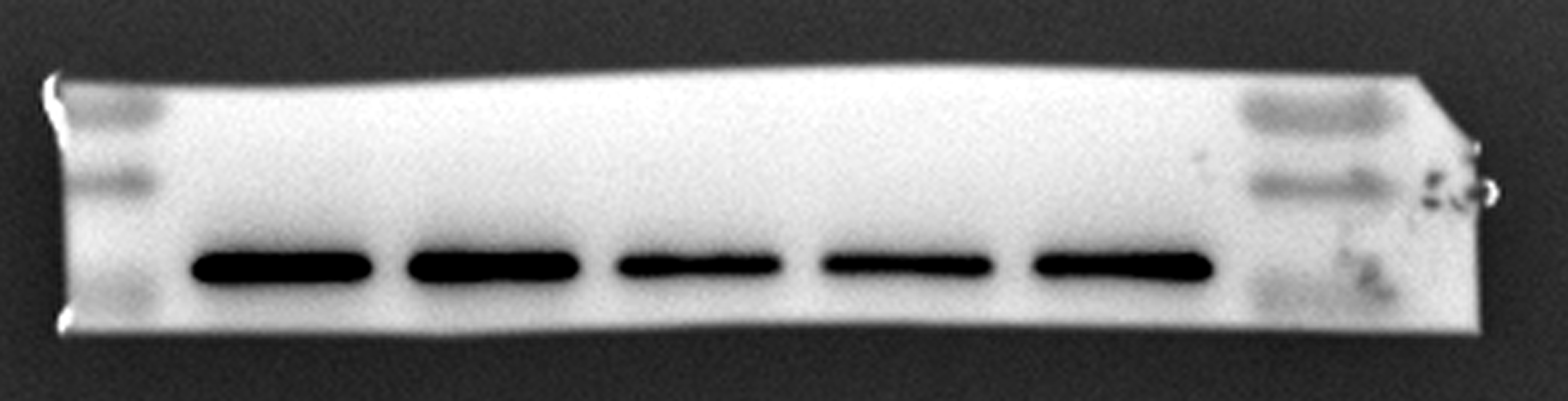

Supplement: Supplemental Material [file KBIE_A_2079253_SM0231.zip › blots/Fig7D/N-cadherin.tif]

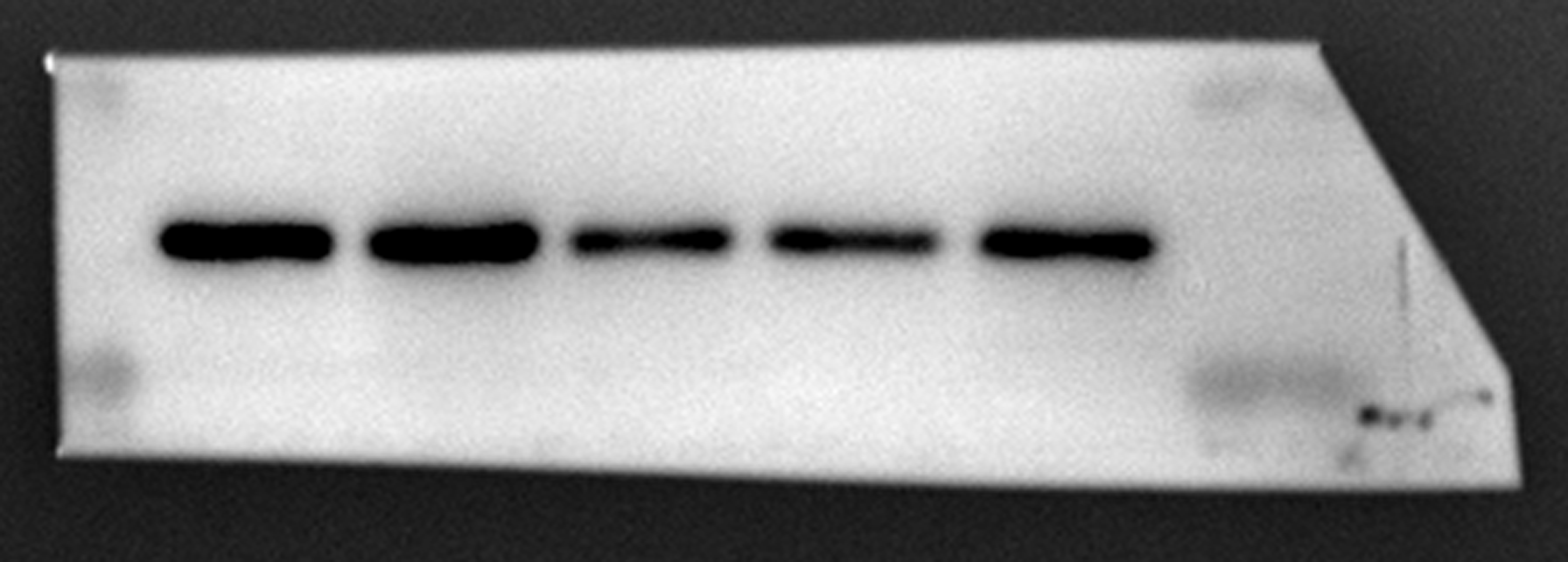

Supplement: Supplemental Material [file KBIE_A_2079253_SM0231.zip › blots/Fig7D/Snail.tif]

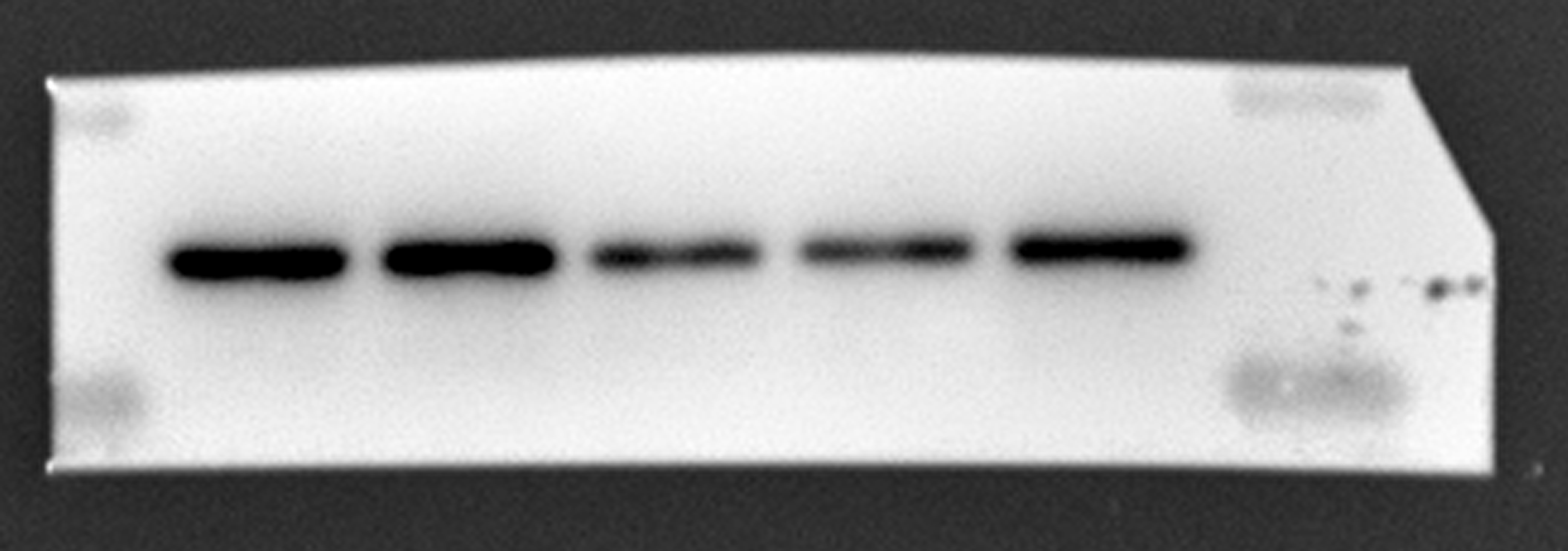

Supplement: Supplemental Material [file KBIE_A_2079253_SM0231.zip › blots/Fig7D/vimentin.tif]

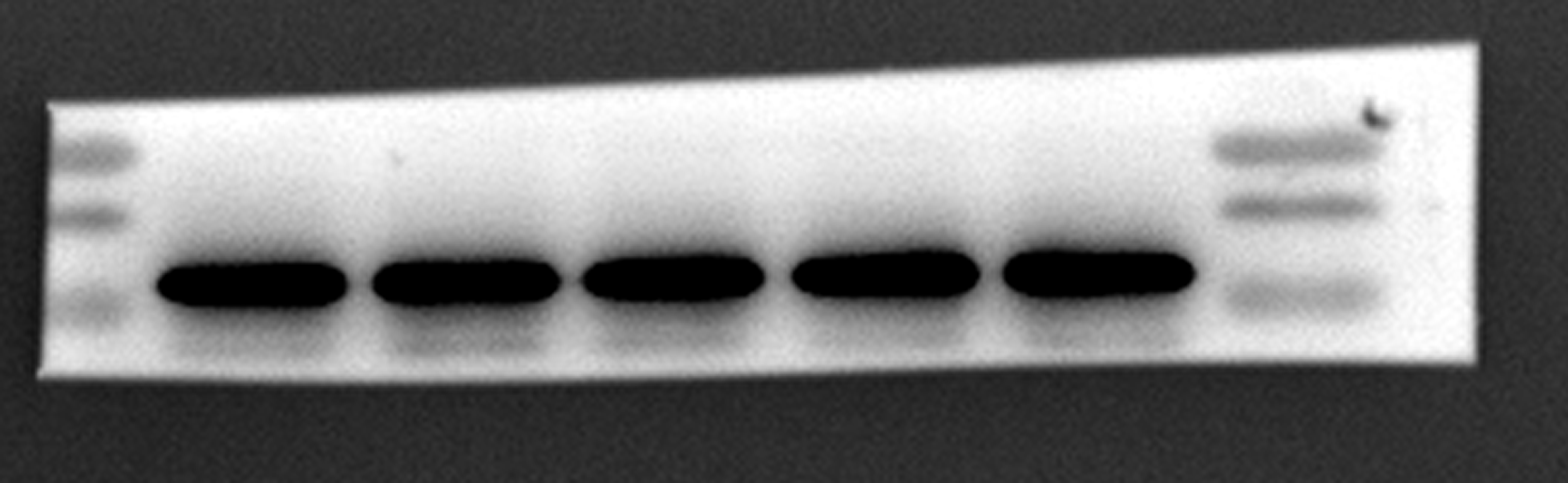

Supplement: Supplemental Material [file KBIE_A_2079253_SM0231.zip › blots/Fig8/AKT.tif]

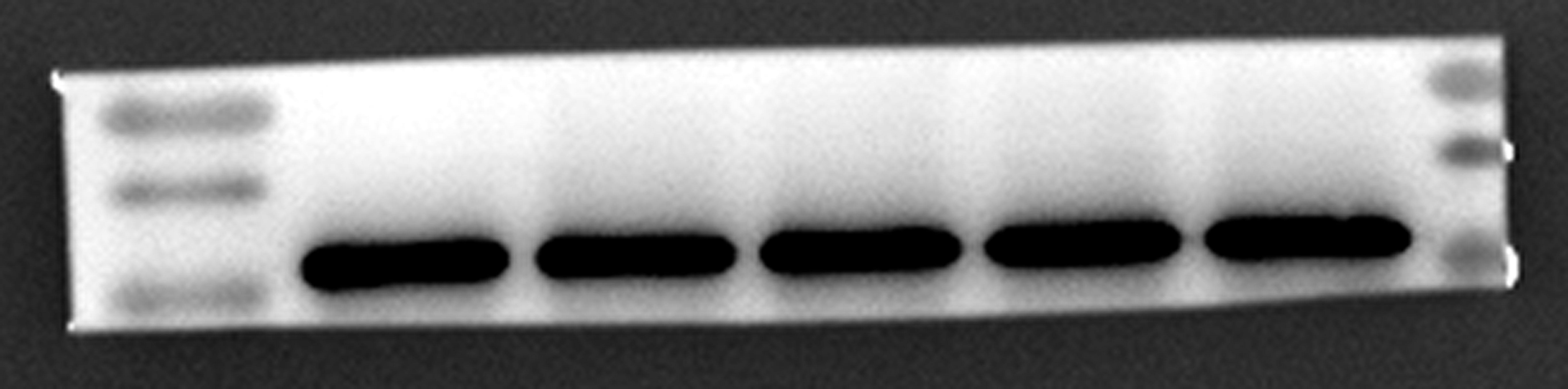

Supplement: Supplemental Material [file KBIE_A_2079253_SM0231.zip › blots/Fig8/gapdh.tif]

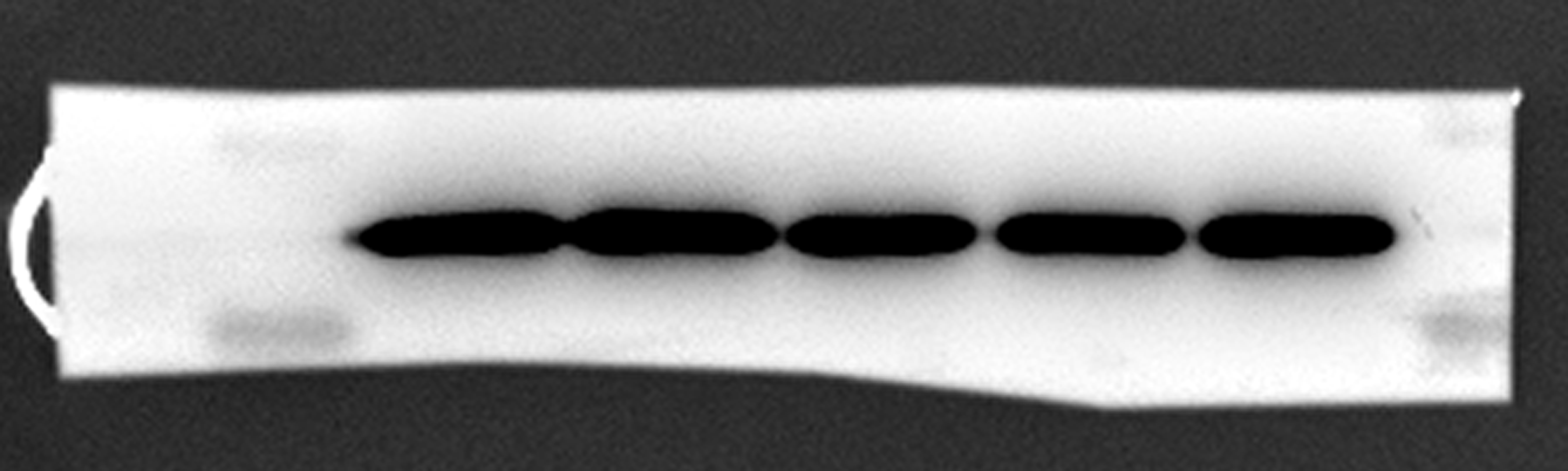

Supplement: Supplemental Material [file KBIE_A_2079253_SM0231.zip › blots/Fig8/GSK3β.tif]

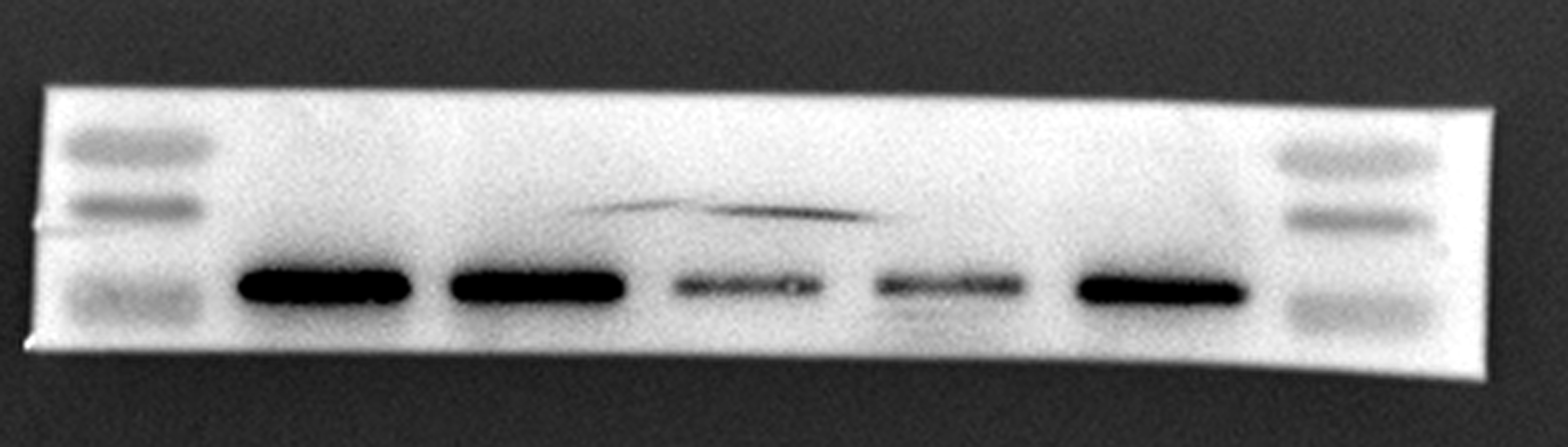

Supplement: Supplemental Material [file KBIE_A_2079253_SM0231.zip › blots/Fig8/P-AKT.tif]

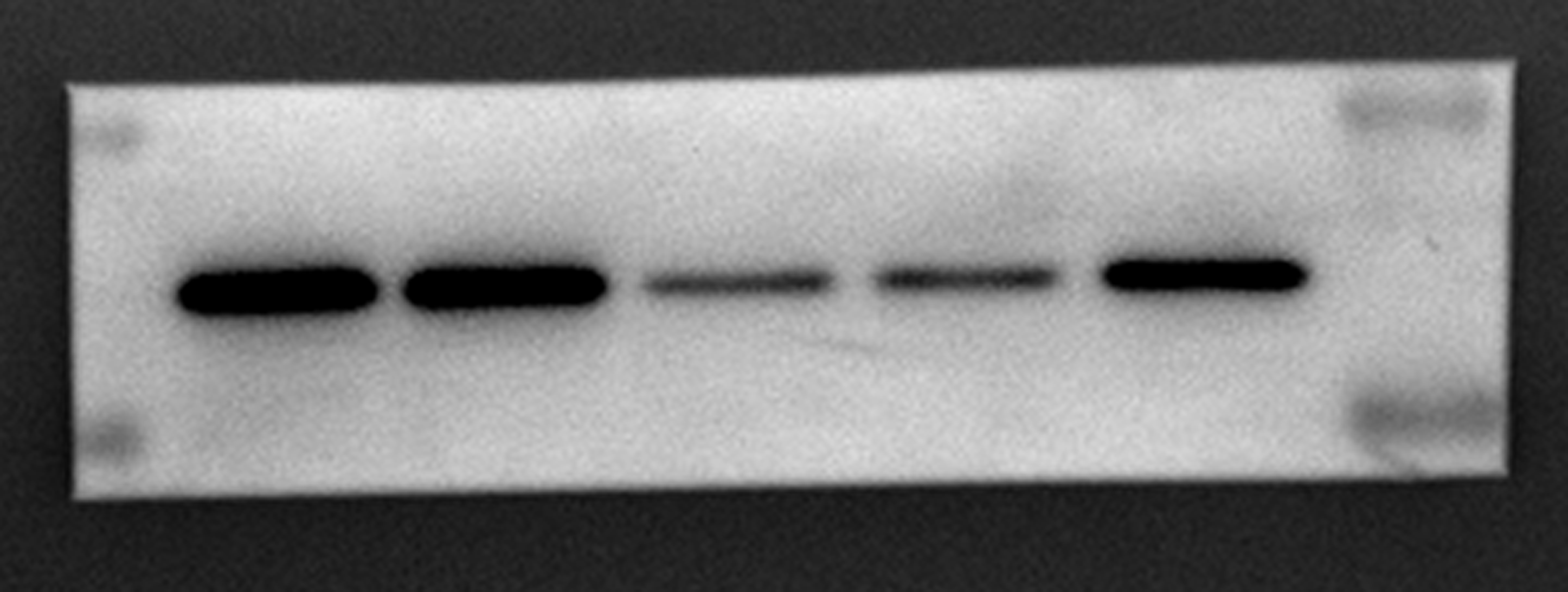

Supplement: Supplemental Material [file KBIE_A_2079253_SM0231.zip › blots/Fig8/p-GSK3β.tif]
